# Small molecules that inhibit TNF signalling by stabilising an asymmetric form of the trimer

**DOI:** 10.1038/s41467-019-13616-1

**Published:** 2019-12-19

**Authors:** James O’Connell, John Porter, Boris Kroeplien, Tim Norman, Stephen Rapecki, Rachel Davis, David McMillan, Tracy Arakaki, Alex Burgin, David Fox III, Tom Ceska, Fabien Lecomte, Alison Maloney, Alex Vugler, Bruce Carrington, Benjamin P Cossins, Tim Bourne, Alastair Lawson

**Affiliations:** 1 UCB Pharma, Slough, SL1 3WE UK; 2 Covance Inc, Princeton, NJ 08540 USA; 3 Broad Institute of Harvard and MIT, Cambridge, MA 02142 USA; 4 UCB Pharma, Bainbridge Island, WA 98110 USA; 5 Present Address: The Institute for Protein Innovation, 4 Blackfan Circle, Boston, MA 02115 USA

**Keywords:** Drug discovery and development, Screening, Structure-based drug design

## Abstract

Tumour necrosis factor (TNF) is a cytokine belonging to a family of trimeric proteins; it has been shown to be a key mediator in autoimmune diseases such as rheumatoid arthritis and Crohn’s disease. While TNF is the target of several successful biologic drugs, attempts to design small molecule therapies directed to this cytokine have not led to approved products. Here we report the discovery of potent small molecule inhibitors of TNF that stabilise an asymmetrical form of the soluble TNF trimer, compromising signalling and inhibiting the functions of TNF in vitro and in vivo. This discovery paves the way for a class of small molecule drugs capable of modulating TNF function by stabilising a naturally sampled, receptor-incompetent conformation of TNF. Furthermore, this approach may prove to be a more general mechanism for inhibiting protein–protein interactions.

## Abstract (web summary)

While biologics have been successfully applied in TNF antagonist treatments, there are no clinically approved small molecules that target TNF. Here, the authors discover potent small molecule inhibitors of TNF, elucidate their molecular mechanism, and demonstrate TNF inhibition in vitro and in vivo.

## Introduction

TNF antagonist treatments have been one of the success stories of recent years. Biologics such as infliximab, etanercept, adalimumab, golimumab and certolizumab pegol have proved efficacious in the clinic and have a well-documented mode of action and side-effect profile^1^. Nonetheless, the use of biologics remains restricted to a subset of the potential patient population, as immunogenicity^2^, supply chain complexity^3^ and health economics restrict their application. It is clear that many more patients could benefit from a small molecule drug with the efficacy of the anti-TNF biologics.

The compound SPD-**304** targets TNF directly and has been shown to inhibit activity by destabilising the TNF trimer, resulting in a dimer that is no longer able to interact with receptor^4^. Although this strategy has so far not led to molecules capable of being developed, work is continuing in this area^5–7^. The TNF trimer may also be induced to disassemble by aggregating small molecules^8^. These, however, behave as promiscuous aggregators^9^ and the inhibition can be reversed with detergent, a hallmark of promiscuous aggregators. Approaches have also been described using natural products such as Japonicone A^10^, and others have targeted the receptor rather than the TNF ligand^11,12^. The status of small molecule inhibitors of TNF has been reviewed in a number of articles^13,14^.

Small molecule modifiers of protein-protein interactions are generally described as orthosteric or allosteric. The former compete directly with the two proteins for binding. There are a number of examples described in the literature^15,16^. The potency of TNF for TNF receptor 1 (TNFR1) is picomolar and despite the interest in the field generated by the success of the biologics, no orthosteric small molecule inhibitors have been described. We believe that disrupting the interaction allosterically^17^ through a region of TNF that is not in direct contact with its receptor may offer advantages that make this an attractive way to block its mechanism of action. In this paper, we describe the discovery and characterisation of a way in which antagonism of TNF signalling is achieved in such a manner with low-molecular-weight compounds. This finding has the potential to broaden the availability of the therapeutic benefits of TNF inhibition.

## Results

### Fragments bind directly to TNF with slow binding kinetics

**Fig. 1:**
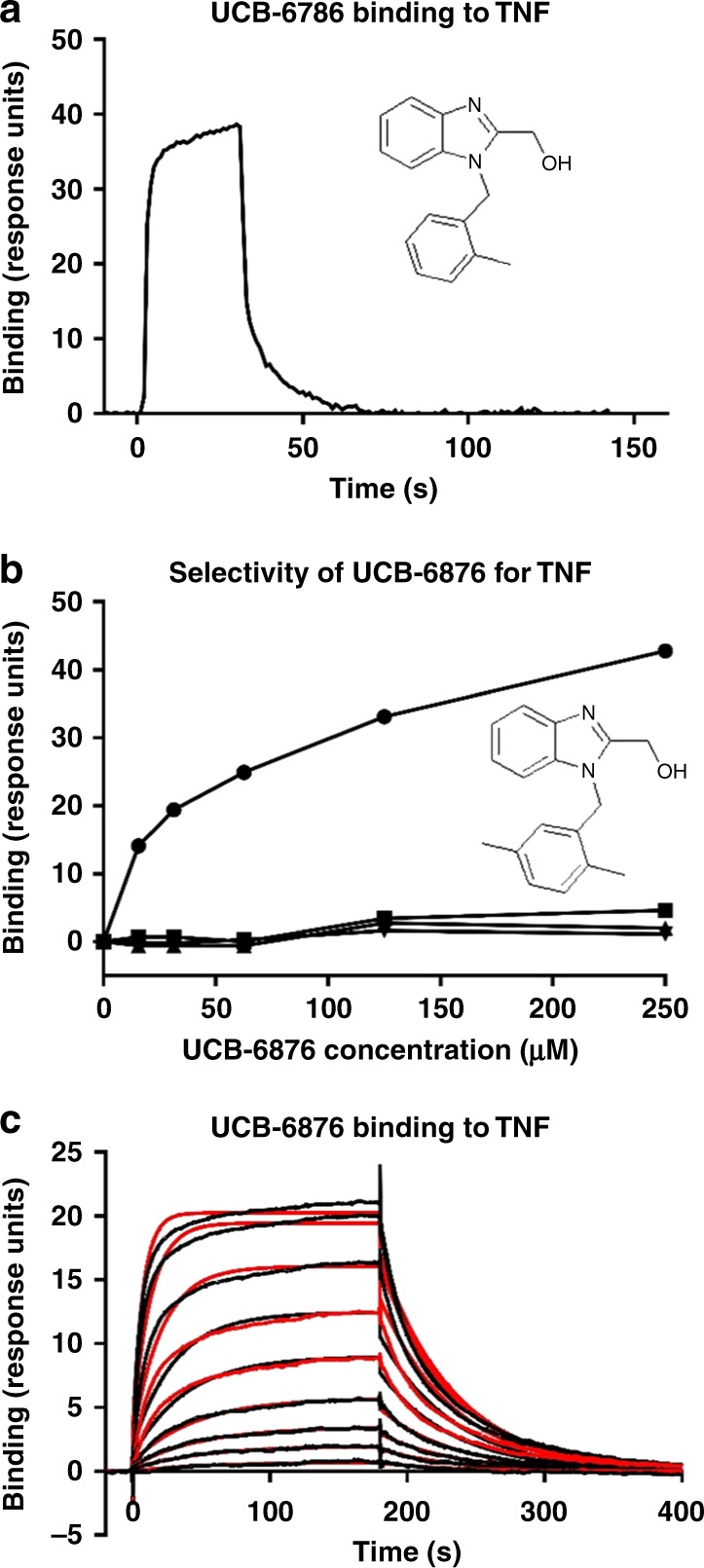
Fragments bind to TNF with slow-binding kinetics. **a** SPR depicts the binding of UCB-**6786** to immobilised TNF. **b** The analogue, UCB-**6876**, specifically binds to immobilised TNF (solid black circles) but not to control proteins, capture antibody plus TNFR1 (solid black squares), capture antibody (solid black triangle pointed down) or TNFR1 (solid black triangle pointed up). **c** Kinetic analysis of UCB-**6876** using the Biacore T100 showing slow association and dissociation rates.

We screened 2000 chemical fragments (MW < 300 Da) using surface plasmon resonance (SPR) measurement on a Biacore 4000. The fragments were passed over the Biacore chip surface coated with immobilised TNF and screened at a concentration of 250 μM to detect weak binders. This screen identified UCB-**6786** (Fig. 1a) and an analogue UCB-**6876**, which bound to TNF displaying a concentration-dependent response curve and selectivity over the TNFR1 extracellular domain and control proteins (Fig. 1b).

We next measured the binding kinetics of UCB-**6876** for TNF by SPR. Association rate constants (k_a_) for typical small molecules are limited by the rate of diffusion and are commonly higher than 10^6^ M^−1^s^−1^ ^18^ but here the k_a_ proved to be remarkably slow (923 M^−1^s^−1^). Conversely, the dissociation rate constant of this compound was also slow, particularly for a fragment (*k*_d_ of 0.02 s^1^) resulting in a *K*_D_ (*k*_d_/*k*_a_) of 22 μM. The slow kinetic profile is unusual for molecules of this size and the resulting curve (Fig. 1c) did not have the square wave shape that is typical of fragment binding.

### Crystal structure and interactions of the TNF: compound complex

**Fig. 2:**
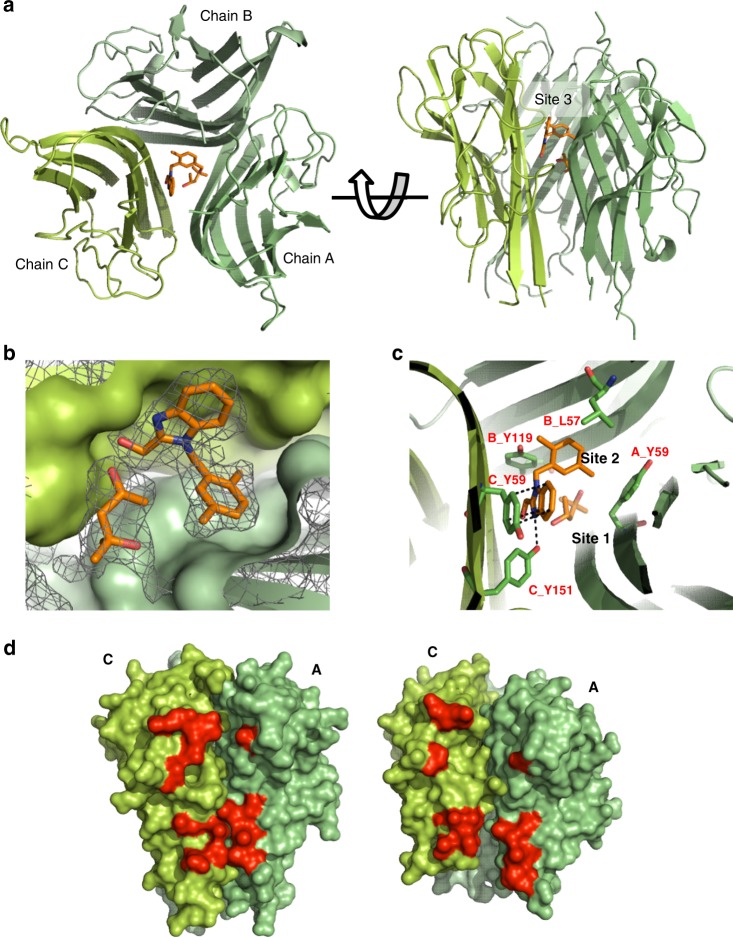
Crystal structure of human TNF with UCB-6876. **a** Top and side views of TNF (green ribbons) with UCB-**6876** bound (orange sticks). **b** Detail showing the electron density of UCB-**6876** and MPD bound within the TNF homotrimer. Monomers B (light green) and C (green) are shown surface rendered. Contour level of the electron density is set at 1 sigma. MPD occupies a space next to UCB-**6876**. Subsequent molecules described in this paper were modified to have chemical groups occupying the space where MPD was bound. **c** Detail of the compound-binding pocket within the TNF homotrimer, with key residues involved in binding highlighted (sticks and labels). **d** Side view of apo-TNF (left image) and UCB-**6876** bound TNF (right image) revealing the distorted AC receptor-binding site (selected residues involved in TNFR1 binding are highlighted red).

Attempts were then made to obtain an X-ray co-crystal structure by soaking apo-TNF crystals with compound and conducting co-crystallisation trials, also in the presence of the compound. In the soaking studies the presence of UCB-**6876**, but not a DMSO mock control, rapidly cracked and eventually dissolved the TNF crystals. In co-crystallisation trials, it prevented the crystallisation of TNF under our standard conditions. After screening for compound-dependent crystallisation conditions, crystals diffracting to 2.5 Å were obtained for UCB-**6876**. The structure was solved by molecular replacement using individual monomers as a search model. The structure shows three TNF monomers in the asymmetric unit (ASU) (Fig. 2a) and clear electron density for one molecule of UCB-**6876** bound at the centre of the TNF trimer (Fig. 2b). The fold of the individual TNF monomers was unchanged compared with apo-TNF (1TNF); however, their spatial arrangement had changed, resulting in a loss of the three-fold symmetry observed in all other published structures of TNF. The compound was buried within the space between the three TNF monomers and did not make any contact with solvent. The key interactions appeared to be the hydrogen bond from a benzimidazole N atom to Y151^C^ (Y151 in chain C) and π stacking of the benzimidazole with Y59^C^. The 2,5-dimethylbenzyl moiety of UCB-**6876** filled a hydrophobic pocket, assigned as Site 2, with compound TNF interactions composed of Y59^A^, Y119^B^ and L57^B^ (Fig. 2c). It is also interesting to note that a single molecule of methylpentanediol (MPD), a co-crystallant required for crystal formation, was observed in an adjacent pocket designated as Site 1 (PDB: 6OOY).

The asymmetry of the compound-bound TNF trimer results in minor distortion at two of the receptor-binding sites and a more significant change at the AC binding site (i.e., the receptor-binding site formed at the interface of the arbitrarily assigned A and C monomers in the crystal lattice). There is a clear opening of the AC site because of monomer A twisting and tilting down and away from monomer C (Fig. 2d). By aligning compound-bound TNF with apo-TNF (1TNF) through monomer C, the degree of movement of selected residues involved in receptor binding on monomer A was measured, revealing shifts of between 5.6 Å and 9.5 Å (Supplementary Table 1 summarises the movement of selected residues and Supplementary Movie 1 uses the crystal structures of apo-TNF and compound-bound TNF to show the overall change in TNF when compound is bound.) Stereo images of portions of the electron density maps can be found in Supplementary Fig. 1.

### Stabilisation of TNF by small molecule binders

**Fig. 3:**
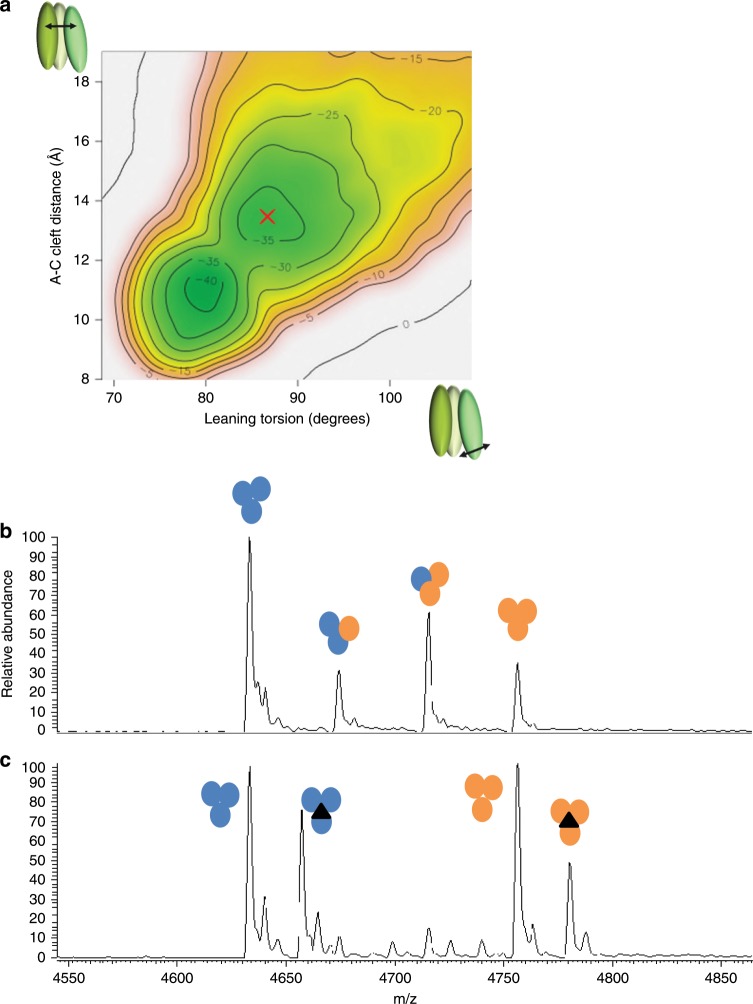
TNF stabilisation by molecular dynamics and monomer exchange. **a** Meta-dynamics molecular dynamics simulation was used to produce this free-energy surface. The simulation used a torsion describing the leaning angle of subunit A with respect to the other subunits and a distance describing how open the AC cleft is. **b** Mass spectrometry analysis of a mixture of human TNF (orange circles) and mouse TNF (blue circles) showing peaks corresponding to homotrimers and heterotrimers. **c** An equivalent analysis using human TNF pre-incubated with UCB-**6876** (black triangle) showing that the compound blocks the exchange of mouse and human monomers.

We were interested in the mechanism of action of these inhibitors and hypothesised that small molecules such as UCB-**6876** are unlikely to induce a large quaternary conformational change as seen here. Also, the UCB compounds bind significantly faster than the timescale suggested for subunit exchange^4^. An enhanced molecular dynamics simulation^19^ was used to investigate the conformational transition between the symmetrical apo-TNF and the asymmetrical, open conformation of the TNF trimer (Supplementary Fig. 2). These simulations were carried out without the inclusion of our small molecule inhibitor in order to understand the stability of the unbound asymmetric open conformation. The free-energy surface (FES) generated by this dynamic model (Fig. 3a) shows two clear energy basins: the symmetrical trimer (at 80 degrees leaning torsion and 11 Å cleft distance) and a stable asymmetric trimer (marked with a red cross). The shape of our FES suggests that the asymmetric open state would be stable without a bound inhibitor, as there is a significant free-energy barrier to returning to the symmetric closed conformation, which is calculated to be about 5 kcal mol^−1^ more stable. Hence, we expect that this new conformation of TNF is formed by conformational selection as suggested by Double Electron-Electron Resonance (DEER) analysis^20^ and stabilised by UCB-**6876**. This model supports our hypothesis that the compounds can bind to and stabilise a naturally sampled transient TNF trimer conformation. That UCB-**6876** binds to a low abundance form of TNF is consistent with the slow on-rate observed by SPR, since the association rate constant is dependent on binding site access and concentration. As indicated by the crystal structure, once bound it is completely buried between the TNF monomers, explaining the slow dissociation rate.

We confirmed that the compounds stabilised the TNF trimer by investigating their effect on the process of TNF monomer exchange. At low physiological concentrations, the trimer can dissociate into monomers^21^, and these have been shown to exchange with monomers from a different TNF molecule^22,23^.

To test this by mass spectrometry, we used mouse TNF, which has a different molecular weight to human and has been shown to exchange monomers by Biacore^22^. We first showed that incubating a mixture of mouse and human TNF quickly produced heterotrimers consisting of different ratios of mouse monomer and human monomer (Fig. 3b). This suggests that the trimer can re-form by exchanging monomers and dimers between mouse and human TNF.

We then saturated human TNF with UCB-**6876** and repeated the experiment. This time only intact human and mouse TNF homotrimers were observed, indicating that significant exchange of monomers between mouse and human TNF had not occurred (Fig. 3c and Supplementary Table 2).

### Improving the affinity of compounds for TNF for in vitro and in vivo studies

To allow us to test for a functional effect in a cell-based assay we set out to improve the affinity of these compounds. Comparison of the co-crystal structure to the symmetrical apo-TNF structure showed that the ligand binding pocket was created by movement of Y119^C^ together with movement of the three TNF monomers relative to one another, creating space for the compound. We extended UCB-**6876** to occupy the MPD binding pocket (termed Site 1 in Fig. 2b) to give the pyridyl analogue UCB-**5307** (Supplementary Fig. 3), which showed a significant improvement in potency (*K*_D_ = 9 nM and *k*_d_ 5.76E−05 s^−1^; Supplementary Fig. 3) over UCB-**6876**. As expected, the co-crystal structure of UCB-**5307** (PDB: 6OOZ) showed that the appended pyridyl group occupied the MPD pocket. The pyridyl nitrogen formed an H-bond with Y119^A^, accounting for the increase in potency (Supplementary Fig. 4). The overall degree of TNF distortion had not changed, however, suggesting that the increased potency is simply due to an increased affinity, hence stabilisation. Indeed, the level of TNF stabilisation measured by thermal denaturation was significantly increased in the presence of UCB-**5307** (Δ11.8 °C) compared with TNF in the presence of DMSO alone (Supplementary Table 3).

We then turned our attention to Site 3, which extends towards the solvent channel. We determined that this would be accessible from the 5-position or 6-position of the benzimidazole. Addition of a pyrazole group at this position produced UCB-**9260** (PDB: 6OP0) (Supplementary Fig. 5), which bound TNF with a similar *K*_D_ of 13 nM but had a slower off-rate in the Biacore assay (*k*_d_ = 4.39E−05 s^−1^; Supplementary Fig. 3), and resulted in further stabilisation of the TNF trimer (Δ17.1 °C) (Supplementary Table 3).

### Consequence of TNF distortion on receptor binding

We were interested in the effect that distortion of the trimer would have on receptor binding and the consequence for signalling activity in cell and animal systems. We used analytical size exclusion (AnSEC) to test if asymmetric TNF was compromised in its ability to bind TNFR1.

**Fig. 4:**
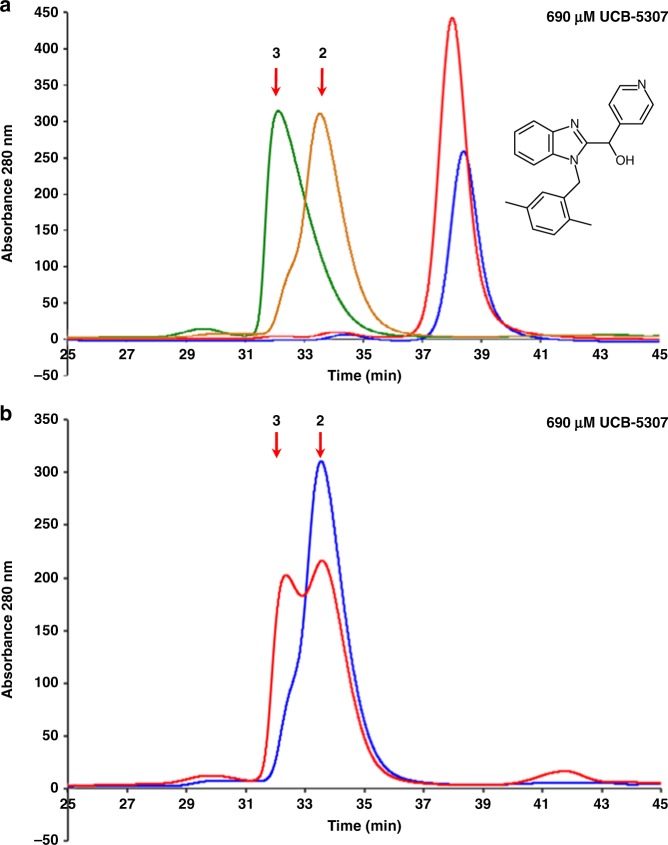
Effect of compounds on TNF:TNFR1 stoichiometry. **a** Analytical size exclusion chromatography (AnSEC) of human (h) TNF alone (blue trace), hTNF + UCB-**5307** (red trace), hTNF + 3.2-fold excess hTNFR1 (green trace) and hTNF + 3.2-fold excess hTNFR1 + 690 µM UCB-**5307** (brown trace). Expected migration position of hTNF with two and three receptors bound are indicated by arrows. Standard trace shown in Supplementary Fig. 5. **b** AnSEC comparing effect of 690 µM UCB-**5307** on preformed hTNF/hTNFR1 complex (3.2-fold excess hTNFR1) (red trace) vs. hTNF preloaded with UCB-**5307** followed by addition of 3.2-fold excess hTNFR1 (blue trace) as in experiment (**a**). Source data are provided as a Source Data file.

To help interpret this, the migration profile of human TNF (hTNF) with one, two or three receptors bound was first characterised by titrating hTNFR1 with hTNF (Supplementary Fig. 6). To measure the effect of compound, hTNF trimer was incubated overnight with four concentrations of UCB-**5307** prior to incubation with a 3.2-fold excess of receptor. The compound had little effect on the migration of hTNF alone (Fig. 4a, red and blue traces), and in the absence of compound, a single peak was observed corresponding to hTNF with three receptors bound (Fig. 4a, green trace). As compound concentration was increased, there was a dose-dependent shift from three receptors bound to two (Supplementary Fig. 7a). At the highest compound concentration (TNF fully occupied by UCB-**5307**) the major species was the two-receptor bound form (Fig. 4a, brown trace).

Preloading hTNF with UCB-**5307** blocked one receptor from binding, so we next tested the ability of UCB-**5307** to disrupt a preformed hTNF/hTNFR1 complex. Complex formed using a slight excess of receptor over TNF was challenged with the same range of compound concentrations, and the results indicate that UCB-**5307** could penetrate the preformed complex, dislodging one of the receptors (Supplementary Fig. 7b). At the highest compound concentration, the degree of disruption was less pronounced compared with that seen when preloading the TNF with UCB-**5307** (Fig. 4b, compare red and blue traces), and the ratio of two receptor-bound to three receptor-bound species was approximately equal (Fig. 4b, red trace).

### TNF small molecule compounds inhibit TNFR1 signalling and downstream function in vitro

**Fig. 5:**
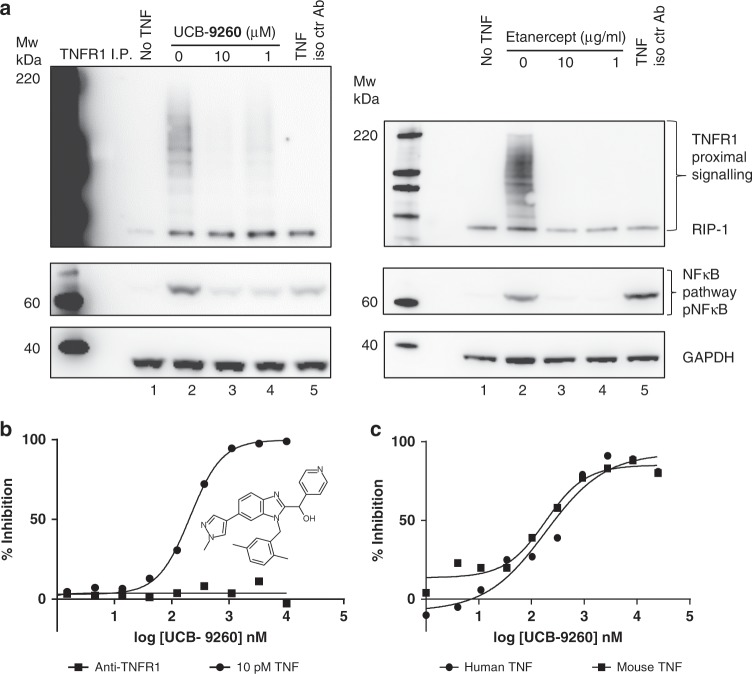
Inhibition of TNF signalling in cells. **a** Western blots measuring RIP1 ubiquitination following TNFR1 immunoprecipitation from Jurkat cells treated with TNF ± UCB-**9260** or etanercept. pNFkB expression was assessed, along with GAPDH for loading control. An isotype control antibody was included as an immunoprecipitation control (lane 5). **b** TNF (10 pM) or anti-TNFR1 agonist antibody (0.3 mg/mL)-driven NFkB activity was measured following UCB-**9260** treatment in Hek-293 cells using a reporter gene assay system. Representative concentration-response curves are shown. Percentage inhibitions for compound dilutions were calculated between a DMSO control and maximum inhibition (by excess biologic anti-TNF, or NFkB inhibitor–TPCA-1). Untransformed data shown in Supplementary Fig. 10c. **c** UCB-**9260** inhibition of TNF (human or mouse) dependent cytotoxicity was measured in mouse L929 cells. Representative concentration-response curves are shown. Percentage inhibition was calculated between unstimulated wells (maximum signal), and wells with TNF, DMSO and actinomycin D (minimum signal). Untransformed data shown in Supplementary Fig. 10d. Source data are provided as a Source Data file.

In order to assess the effect of the compounds in the TNFR1 signalling pathway, we first looked at events proximal to TNFR1 by measuring signalling protein recruitment to TNFR1, in particular RIP-1, which appears as a ubiquitinylated ladder on gels^24^. In addition, we evaluated the downstream signalling endpoint, phosphorylated NF-κB (pNF-κB), which is distal to the TNF:TNFR1 signalling complex^25^. After TNF stimulation alone, increased levels of RIP were recruited to TNFR1, with a concomitant increase in phosphorylation of NF-κB (Fig. 5a, lane 2). However, when TNF was pre-incubated for 1 h with UCB-**9260**, recruitment of these proteins to the TNFR signalling complex, and subsequent phosphorylation of NF-κB, were markedly reduced to levels approaching those seen when TNF was pre-incubated with the TNF biologic, etanercept (Fig. 5a). This demonstrated that these compounds inhibited the ability of TNF-stimulated TNFR1 to recruit downstream proteins involved in signalling through NF-κB. We next determined the cell potency and selectivity of compounds in a high-throughput human cell-based reporter gene assay measuring the response of SEAP-linked NF-κB to TNF (human or mouse) stimulation in the HEK 293 cell-line. In the same system, we were also able to test the selectivity to TNF by using an agonistic monoclonal antibody to TNFR1 (0.3 μg/mL) to stimulate NF-κB via the same pathway (Supplementary Fig. 8). UCB-**9260** inhibited NF-κB with a geometric mean IC_50_ of 202 nM (95% CI = 53, *n* = 8) after TNF stimulation (Fig. 5b Supplementary Fig. 9b), but there was no inhibition of NF-κB stimulated by the TNFR1 agonist antibody. Since both TNF and the agonistic antibody signal via the same pathway this indicates that the effect of UCB-**9260** is through TNF in this system.

We then tested if the compounds could inhibit both human and mouse TNF-dependent functional activity by evaluating UCB-**9260** using a mouse cell L929 TNF-dependent cytotoxicity assay, which had previously been used to characterise TNF biologics in vitro^26^. UCB-**9260** inhibited TNF-dependent cytotoxicity with a geometric mean IC_50_ of 116 nM, (95% CI = 15, *n* = 203) and a geometric mean IC_50_ of 120 nM, (95% CI = 16, *n* = 180) using human and mouse TNF, respectively (Fig. 5c and Supplementary Fig. 9b). These data showed that the compound inhibited the signalling and function of TNF and was suitable for testing in mouse in vivo models.

### Mouse in vivo activity

Since UCB-**9260** was to be used for in vivo studies, we first confirmed that there were no selectivity flags using the CEREP ExpresSProfile panel. We then tested the effect of this compound on other members of the TNF superfamily with trimeric structures similar at a superficial level at least to TNF. We used thermal melting to test the ability of UCB-**9260** to stabilise other members of the TNF superfamily in the presence of DMSO compared with TNF (Supplementary Figs. 10, 11). No other protein tested showed a significant change in Tm, suggesting that the action of UCB-**9260** is specific to TNF.

We were also interested in trying to understand why these molecules were so selective for TNF over other family members. This was particularly surprising because some of the family members have similar residues to TNF at the binding site. We tested one possibility: that compounds could not access the binding site in the centre of the trimer of these proteins. We noted by comparing crystal structures that TNF had a leucine at position 57, whereas many of the other members of this family had a larger phenylalanine or tyrosine. This is close to the binding pocket, and in the symmetrical molecules these residues are close to one another (one from each chain) thus forming a ‘gateway’ to the pocket. Accordingly, we mutated Leu57 of TNF to phenylalanine, mimicking other family members, to which our compounds did not bind in a thermal melt assay. The mutated TNF (L57F) was equally active as wild-type TNF in a HEK assay (Supplementary Fig. 9c), but the compounds showed no activity as demonstrated with UCB-**9260** (Supplementary Fig. 9d).

**Fig. 6:**
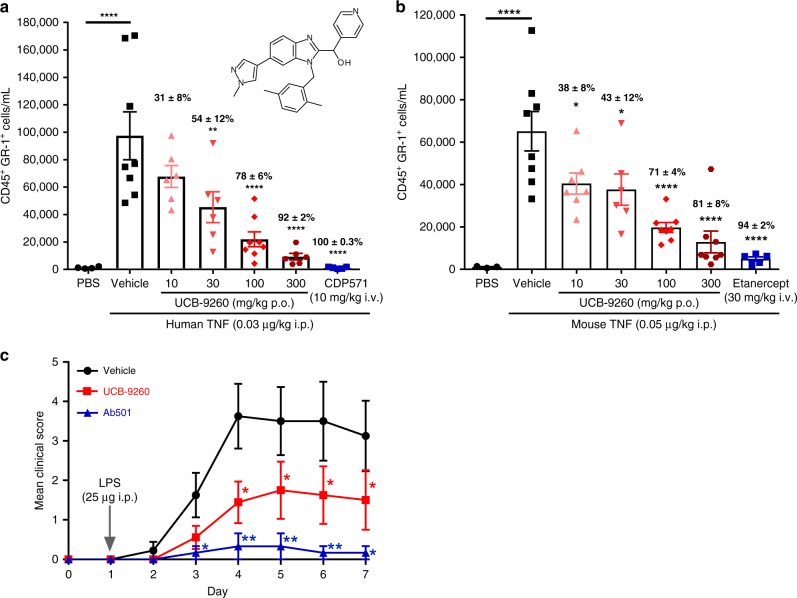
Oral treatment with UCB-9260 inhibits TNF-driven functional effects in vivo. **a** UCB-**9260** (10–300 mg/kg po, PBS *n* = 4; vehicle *n* = 8; UCB-**9260** 10, 30 and 300 mg/kg *n* = 6 and 100 mg/kg *n* = 8; CDP571 *n* = 5 mice) dose-dependently inhibits human and (**b**) mouse TNF-induced neutrophil recruitment to the peritoneal compartment in mice (PBS *n* = 3; vehicle *n* = 8; UCB-**9260** 10 mg/kg *n* = 7, 30 mg/kg *n* = 6, 100 and 300 mg/kg *n* = 8; CDP571 *n* = 5 mice). **c** UCB-**9260** (150 mg/kg po bid) significantly reduces arthritis clinical score in the CAIA model (vehicle *n* = 8; UCB-**9260** *n* = 8 and AB501 *n* = 6 mice). Mean data ± standard error is shown. *p < 0.05, **p < 0.01, ***p < 0.001 determined using one-way ANOVA with Dunnett’s multiple comparison test. Exposure of UCB-**9260** can be found in Supplementary Tables 4–6. Source data are provided as a Source Data file.

Having confirmed that UCB-**9260** was selective for TNF over other superfamily members, it was tested for activity in vivo using a TNF-dependent mouse model. One such model measures the ability of exogenous TNF injected into the mouse peritoneal cavity to induce neutrophil recruitment^27^. Either human or murine TNF can be tested because both signal through murine TNFR1^28^. We performed a dose-response experiment with UCB-**9260** to assess its ability to inhibit both TNF stimuli, compared with a suitable anti-TNF biologic (CDP**571**)^29^ to inhibit human TNF, and etanercept to inhibit murine TNF. Following oral administration, UCB-**9260** showed a dose-dependent inhibition of neutrophil recruitment, measured as CD45^+^GR1^+^ cells by flow cytometry with significant inhibition obtained at 30 mg/kg of human TNF (Fig. 6a) and significant inhibition of murine TNF after a 10 mg/kg dose (Fig. 6b and Supplementary Table 4). Additional supporting data using a zymosan-induced neutrophilia model can be found in Supplementary Fig. 12 and Supplementary Table 5.

The collagen antibody induced arthritis (CAIA) model uses a combination of an anti-collagen antibody cocktail and lipopolysaccharide (LPS) to produce rheumatoid arthritis-like symptoms in the mouse^30^. In this model, an in-house blocking murine antibody (Ab501) showed profound inhibition of the clinical score (Fig. 6c). UCB-**9260**, dosed orally at 150 mg/kg twice daily (bid), 6 h after the LPS challenge (therapeutic dosing), also showed a significant reduction of the clinical score (Fig. 6c). Statistically significant inhibition in these models was associated with unbound blood concentrations of compound that were maintained above the in vitro human and mouse TNF IC_50_ values (Supplementary Table 6).

## Discussion

We have discovered orally active small molecules that inhibit TNF activity both in vitro and in vivo. The compounds bind to a pocket in the centre of the TNF trimer formed by the movement of the TNF monomers, stabilising the distorted trimer, which leads to reduced signalling through TNFR1. Analytical size exclusion suggests that the asymmetric trimer only binds to two receptors, consistent with the structural data showing significant distortion at one receptor binding interface. Rather than inducing a conformational change, we believe that the compounds bind to TNF through a process that is largely governed by conformational selection as suggested by our molecular dynamic (MD) simulations (Fig. 3a) and DEER experimental data. We show here an approach to inhibiting TNF through allosteric binding using small, drug-like, orally available molecules that we believe stabilise a naturally occurring conformer of TNF.

Inhibiting protein-protein interactions (PPIs) with small molecules has been described as reaching for the ‘high hanging fruit’^31^. Indeed, the disruption of PPIs has been considered difficult by medicinal chemists for many years and there are few examples in the literature^32^. The surface between proteins is typically large, flat and featureless. Accordingly, many groups choose to target these interactions using relatively large molecules based on peptides or scaffolds that mimic peptides. Other approaches, however, have been described, such as targeting ‘hot spots’ in the binding site that appear to contribute most of the binding energy, making smaller molecules a viable alternative^33^. Notably, so-called fracturing of CD40L, a member of the TNF superfamily, using a relatively large molecule retained the trimeric assembly^34^. Indeed, there is some overlap between where this compound binds on CD40L and the TNF binders described here. Furthermore, stabilisation of protein complexes by binding naturally occurring small molecules at the interface has also been described^35^, and it is interesting to speculate that there could be naturally occurring molecules that specifically modulate the function of TNF. Most importantly, this work suggests a method of discovering inhibitors using protein stabilisation of a non-active conformation as an end point, which could be facilitated by an antibody that holds the target protein in the desired inactive conformation^36^. Here we demonstrate that fragment-based drug discovery can be used to provide starting points for orally active compounds that modulate protein-protein interactions.

Small molecules offer several advantages over biologics, including cost, the convenience of oral delivery and shorter half-lives, which benefit patients requiring drug holidays. In addition, they can be designed to diffuse across the blood-brain barrier, making them suitable for targeting central nervous system disorders. The possibility of being able to target TNF-driven neuroinflammation, for example, is particularly interesting.

From our experience with TNF, it seems that orally available, bona fide small molecules, capable of stabilising favoured, naturally sampled conformations of target proteins, offer an attractive and chemically tractable approach to tackling protein-protein interactions.

## Methods

### Biacore binding assay

BIA (Biomolecular Interaction Analysis) uses SPR to measure the binding of compounds to a protein immobilised on a chip surface. For this purpose a Biacore A100/4000 (GE Healthcare) was used. A CM5 sensor was immobilised via amine coupling on flow cell 1 with human Fc antibody domain (10k response units; kRU) and TNFR-Fc captured (2.5 kRU), flow cell 2 TNF (11 kRU), flow cell 3 was left blank (for non-specific binding), flow cell 4 TNFR-Fc (10 kRU), and flow cell 5 goat anti-human Fc 16 kRU). The sensor surface was then equilibrated in HBS-P (10 mM HEPES pH 7.4, 0.15 M NaCl, 0.005% Surfactant P20, BIAcore, GE Healthcare) with 5% DMSO for at least 5 h. Compounds were diluted from 100 mM stocks into DMSO matched buffer and then flowed over the immobilised flow cells at a concentration of 250 µM at the maximum flow rate of 30 μL/min. Background subtraction binding curves were analysed using the Biacore A100 BIA evaluation software version 1.1 following standard procedures.

### Biacore kinetics of binding

Surface plasmon resonance using a Biacore T100/T200 was used to measure the association rate constant (ka), the dissociation rate constant (kd) and the affinity (K_D_) of compounds. TNF (VCID 2043 Beryllium) was immobilised to approximately 5 kRU as described above and equilibrated for at least 5 h in HBS P + buffer with 5% DMSO. UCB-**6876** was serially diluted from 187.5 µM, flowing over the immobilised TNF at 50 µL/min for 3 min before allowing full dissociation under flow for at least 6 min. Kinetic constants were obtained from multicycle data that had been double-referenced, the data fitted and then analysed using the Levenberg Marquardt algorithm in the BIA Evaluation software (Biacore T100 BIA evaluation version 1.1). Compounds UCB-**5307** and UCB-**9260** were titrated from 30 µM with three-fold serial dilutions to 0.37 μM and flowed over approximately 1.5 kRU of linked TNF with an n-terminal HKH tag (Beryllium) in HBS P buffer with 5% DMSO at 100 μl/min. The compounds were allowed to dissociate for 2 h under flow. Kinetic constants from single-cycle data were obtained (Biacore T100 BIA evaluation version 1.1) and compound characterisation details can be found in Supplementary Notes 1–4.

### Crystallography: plasmid construction

Human TNF (CID2043, UniProt P01375 residues 77–233 [full plasmid sequences available in the source data file]) was codon-engineered in-silico using GeneComposer™ for *E. coli* expression, and optimised to balance GC content, exclude cryptic Shine Dalgarno sequences, as well as exclude BamHI and HindIII restriction sites. The final gene insert was flanked with 5′ GGATCC (BamHI) and 3′ TGATAAGCTT (HindIII is underlined), such that two stop codons follow the C-terminal residue. The final gene insert was then synthesised by DNA 2.0 and delivered in a shuttle vector. Following synthesis, the gene insert was digested with BamHI and HindIII and subcloned to vector pEMB54, which is an ampicillin-resistant, arabinose-inducible vector with pMB1 origin of replication and 6XHis-Smt3 under the PBAD promoter, followed by a multiple-cloning site which includes BamHI followed by HindIII. After BamHI/HindIII cloning into pEMB54, gene inserts are fused in-frame with the 6XHis-Smt3 sequence. Following digestion of both pEMB54 and CID2043 inserts with BamHI/HindIII, both were gel-purified, the inserts ligated into the vector and the ligation transformed to chemically competent TOP10 cells. One transformant was mini-prepped and submitted for DNA sequencing of the Open Reading Frame. CID7210 (see source data for plasmid sequence) was cloned in a manner similar to CID2043, and consisted of a triple tandem fusion of the TNF ECD (‘TNF Trimer’), wherein Uniprot P01375 77–233 was followed by 85–233, with an SG linker between the TNF monomers. This gene (identical to CID3747) was optimised in GeneComposer with identical BamHI/HindIII adaptors, synthesised by DNA 2.0 and subcloned into final expression vector pEMB116, which is the same as pEMB54 except the N-terminal tag is HKH instead of 6XHis-Smt3. CID8703 was cloned via Quikchange mutagenesis (Agilent) using standard protocols, using CID2043 as a template.

### Crystallography: protein expression and purification

Briefly, the target-specific vector was transformed into TOP10 *E. coli* cells. A starter culture containing 100 µg/mL (final concentration) ampicillin (Teknova) was inoculated with a single colony and grown for 16 h at 37 °C. This was then transferred to 8 L of Terrific Broth (Teknova) containing 100 µg/mL (final concentration) ampicillin and grown to OD600 = 0.6. Protein expression was induced by adding arabinose to a final concentration of 0.1% (VWR) and grown for 16 h at 25 °C. The cells were harvested by centrifugation (Beckman) at 6240×*g* for 15 min and the pellets were collected and stored at −80 °C.

Cells were resuspended 1 g:4 mL in 25 mM Tris(hydroxymethyl)aminomethane hydrochloride (Tris-HCl) pH 8.0 (Teknova), 200 mM NaCl (Teknova), 0.02% 3-[(3-Cholamidopropyl)dimethylammonio]-1-propanesulfonate (CHAPS) (JT Baker), 50 mM L-arginine (Sigma), 500U of benzonase (Novagen), 100 mg lysozyme (Sigma) and one complete EDTA-free protease inhibitor tablet (Roche). The cells were lysed via sonication (Misonix) and clarified via centrifugation at 142,000×*g* for 30 min at 4 °C (Beckman) and filtered with a 0.2 µm bottle-top filter (Nalgene). The supernatant was applied to two 5 mL Ni^2+^ charged HiTrap Chelating HP (GE Healthcare) columns and the protein eluted with a 500 mM imidazole gradient over 20 column volumes. The fractions of interest were pooled and the His-Smt tag was removed via cleavage with Ubiquitin-like-specific protease 1 (Ulp-1) while dialysing against 2 L of 25 mM Tris pH 8.0 and 200 mM NaCl overnight at 4 °C using 3.5 kDa MWCO snakeskin dialysis tubing. The affinity tag was removed by applying the digested pool over two 5 mL Ni2 + charged HiTrap Chelating HP columns. The flow-through contained the cleaved protein of interest. The protein was concentrated for size exclusion chromatography via centrifugal concentration (Vivaspin Polyethylsulfone, 10 kDa MWCO, Sartorius) to 13.5 mg/mL for injection over a HiPrep 16/60 Sephacryl S-100 HR (GE Healthcare) in 10 mM HEPES (2-[4-(2-hydroxyethyl)piperazin-1-yl]ethanesulfonic acid), pH 7.5 and 150 mM NaCl. Fractions of interest were pooled and concentrated via centrifugal concentration (Vivaspin Polyethylsulfone, 10 kDa MWCO, Sartorius) to 20 mg/mL, aliquoted and stored at −80 °C.

### Crystallography: crystallisation

Purified human TNF was diluted to 4–7 mg/mL in 10 mM HEPES pH 7.5, 150 mM NaCl buffer followed by overnight incubation at 4 °C with 0.5 mM compound (1–2 molar excess). The TNF-compound complex was crystallised by sitting drop vapour diffusion by mixing 0.5 μL of complex with 0.5 μL of 13% MPD, 13% PEG 1000, 13% PEG3350, 0.1 M HEPES, pH 8.0. Crystals were harvested for data collection ~2 weeks after initial set-up. Crystals were cryo-protected in ethylene glycol and/or vitrified directly in liquid nitrogen for data collection.

### Crystallography: structure determination

The dataset for human TNF complexed with UCB-**6876** and UCB**-9260** were collected at the Stanford Synchrotron Radiation Lightsource (SSRL) beam line 7–1 (ADSC Quantum-315R CCD X-ray detector). The dataset for human TNF complexed with UCB**-5307** was collected at the Advanced Light Source (ALS) beam line 5.0.3 (ADSC Quantum-315R CCD X-ray detector). Diffraction data were reduced and scaled with XDS/XSCALE^37^. The structure was solved by molecular replacement using Phaser with input model of 1TNF. Iterative manual model building using Coot (Emsley and Cowtan, 2004)^38^ and Refmac (Murshudov et al., 1997)^39^ continued until R and Rfree converged. Model quality was validated using Coot and MolProbity^40^. Structures were validated using Molprobity prior to deposition in the Protein Data Bank (PDB: 6OOY, PDB: 6OOZ, PDB: 6OP0)^41,42^. Statistics for each crystal structure are provided in Supplementary Table 7.

### Mass spectrometry monomer exchange

Mouse and human TNF protein were made up to a concentration of 2 mg/mL (20 mM ammonium acetate, pH 7.4) and desalted and buffer-exchanged using Zeba columns. The protein was then diluted to 20 μM in ammonium acetate buffer. An aliquot of each was mixed 1:1 and analysed at various time points. Human TNF was incubated (1:1, v/v) with compound (20 μΜ), 4% DMSO in 20 mM ammonium acetate) and mixed with mouse TNF (1:1) and tested at various time points using an Advion Nanomate (for sample introduction by nanospray) and ThermoFisher Exactive Plus EMR Mass Spectrometer. The following settings were utilised on the mass spectrometer: scan range 1000 to 8000 *m*/*z*; spray voltage 1.5 kV; capillary temperature 150 °C; S-lens RF 200; source DC offset 25 V, injection flatapole 7 V; inter flatapole 6 V; bent flatapole DC 6 V; EMR mode on. Data were processed using ThermoFisher Protein Deconvolution 3.0.

### Molecular dynamics simulation

Missing backbone atoms were added using FREAD^43^ database structure prediction software. An error in the amino acid sequence of TNF structure 1TNF was detected within this process. In all three monomers of PDB structure 1TNF residue 143 is a leucine; this is in contrast to sequence data for TNF stored in Uniprot^44^ where this residue is an aspartate. For this study residue 143 of 1TNF was mutated to an ASP. Maestro^45^ was then used to add all other missing atoms and pick sensible protonation states for titratable residues, ensuring that all homotrimers remained symmetric.

The ff99SB^45^ Amber force field was employed for all protein parameters, set up using the LEaP software from AmberTools 1.4^46^. The TIP3P^47^ water model was used in a solvating cube along with neutralising sodium chloride ions at an approximate concentration of 0.15 M. NAMD 2.7^48^ was used to carefully equilibrate for temperature and pressure (300 K, 1 atm). ACEMD^49^ was then used for a 1 ns structural equilibration and when patched with Plumed 1.3^50^, for all metadynamics simulations. ACEMD simulations used a 4 fs time step throughout. All simulations used particle mesh Ewald^51^ for long-range electrostatics with appropriate potential truncation and smoothing.

Initial metadynamics simulations used two CVs based on a quaternary torsion and a distance (Supplementary Fig. 1c, d). Gaussians were added every 1 ps with an initial height of 0.1 kcal mol^−1^. The bias-factor was set to 15. Gaussian widths were set on a system basis using the last 200 ps of a 500 ps test run to estimate average fluctuation seen in 1 ps for each CV. A Gaussian width was chosen to be less than half of the average 1 ps fluctuation. Potential walls were employed to stop the opening subunits from dissociating. In these cases the inversion conditions of Crespo et al. were used to limit any error in the FES^52^. Wall and inversion parameters were chosen in line with the Gaussian widths being used. Convergence was pre-defined as a Gaussian height <100 times smaller than the initial value, convergence of any FE difference between open and closed states and free movement across any energy barriers. Hence, once a significant low energy region is found away from the apo (symmetric homotrimer state) region, a return to the apo region was deemed necessary.

PCA analyses of initial metadynamics simulations were carried out using the GROMACS^53^ programmes g_colvar and g_anaeig. The most important eigenvectors were chosen using the relative size of their eigenvalues.

### Thermal denaturation assay of thermal stability

A fluorescence probed thermal denaturation assay was performed to assess the effect of the compounds on the thermal stability of TNF (or other members of the TNF family) as a measure of compound binding. The reaction mixture contained 5 µL of 30× SYPRO^®^ Orange dye (Invitrogen) and 5 µL of TNF (at 1 mg/mL), 37.5 μL PBS, pH 7.4 and 2.5 μL of compound (at 2 mM in DMSO). The mixture (10 µL) was dispensed in quadruplicate into a 384 PCR optical well plate and was run on a 7900HT Fast Real-Time PCR System (Agilent Technologies). The PCR System heating device was set at 20 °C to 99 °C with a ramp rate of 1.1 °C min^−1^; fluorescence changes in the wells were monitored by a CCD device. The fluorescence intensity increase was plotted as a function of temperature and the *T*_m_ calculated as the midpoint of this denaturation curve (determined as the point of inflection).

### Analytical size exclusion chromatography

A stock of compound bound to human (h) TNF was prepared by pre-incubating hTNF (300 μM, molecular weight based on the homotrimer) overnight at 4 °C with UCB-**5307** at a final concentration range from 90 μM to 690 μM, maintaining a constant DMSO concentration of 1%. The compound-bound hTNF was incubated for 1 h at 22 °C with hTNFR1 at a 3.2-fold molar excess over homotrimers (final concentrations: hTNFR1 240 μΜ, hTNF 75 µM). Identical samples were prepared with DMSO only, giving a final DMSO concentration of 0.25% for both sets of samples. Alternatively, a fully assembled hTNF/hTNFR1 complex (3 receptors per homotrimer) was formed by incubating hTNF with a 3.2-fold molar excess of hTNFR1 for 1 h at 22 °C. The TNF/TNFR1 complex was then incubated overnight at 4 °C with UCB-**5307** at a final concentration range from 90 µM to 690 µM. Samples were subject to analytical size exclusion using HPLC. Injection volumes of 50 µL were separated on a TSK G3000SW L × I.D. 30 cm × 7.5 mm column (10 µm particle size) pre-equilibrated in 10 mM HEPES, pH 7.5, 150 mM NaCl.

### TNFR1 immunoprecipitation and signalling assay

TNF (25 ng/mL) was pre-incubated with UCB-**9260** (10 µM) or anti-TNF (20 µg/mL, Etanercept, GlobalRx) for 1 h at 37 °C, and then incubated for 5 min at 37 °C with 50 × 10^6^ Jurkat cells. Cells were then lysed for 1 h in cold lysis buffer (1% NP-40, 150 mM NaCl, 50 mM Tris pH 8.0, 25 mM NaF, 1 mM vanadate, 1% phosphatase inhibitor and 0.2% protease inhibitor cocktail), and cell supernatant harvested. TNFR1 immunoprecipitation was performed by incubating supernatant with 5 µg anti-TNFR1 antibody (R&D systems, #AB-225-PB) overnight while rotating at 4 °C, followed by the addition of 20 µL protein G sepharose beads for 1 h rotating at 4 °C. An isotype control sample was incubated with 5 µg goat IgG, instead of anti-TNFR1 antibody to assess how much unspecific binding to the beads occurs during immunoprecipitation. Beads were subsequently washed 3× in lysis buffer, and then the supernatant removed and SDS-PAGE reducing sample buffer added to beads and samples boiled for 10 min. Sample (15 µL) was then loaded on a 4–12% Bis-Tris gel and run for 65 min at 200 V in MOPS buffer. Gels were then transferred to nitrocellulose membrane using iBlot (Invitrogen) and blocked in 5% milk in TBS/0.05% Tween (blocking buffer) for 1 h. RIP-1 ubiquitination (1:250 dilution, BD, #610459) was then detected by Western blotting using anti-mouse-HRP (1:2000 dilution, Cell Signalling Technology, #7076). pNFκB (1:1000 dilution, Cell Signalling Technology, #3033) and GAPDH (1:4000 dilution, Cell Signalling Technology, #5174) were measured in the total cell lysates by Western blotting using anti-rabbit HRP (1:2000 dilution, Cell Signalling Technology, #7074). Blots were developed by detecting chemiluminescence using ImageQuant (GE Healthcare).

### TNF reporter gene assay using a HEK Blue Media^TM^ readout

Stimulation of HEK-293 cells by TNF leads to activation of the NF-κB pathway. The HEK-Blue^TM^ CD40L SEAP (secreted embryonic alkaline phosphatase) reporter cell line used to determine TNF activity (InvivoGen, #hkb-CD40, cells were described as mycoplasma free when purchased and were tested using Mycoalert [Lonza] after each batch of assay vial preparation). Compounds were titrated in DMSO and pre-incubated with TNF at a concentration giving half maximal response (10 pM for human or 20 pM for mouse) or the anti-human TNFR1 agonist antibody (R&D Systems. #AF 225) for 1 h. Cells were added to the compound/stimulus mixture and further incubated for 18 h. SEAP was measured using the colorimetric substrate HEK-Blue Detect^TM^ or QUANTI-Blue^TM^ from InvivoGen. Percentage inhibitions for compound dilutions were calculated between a DMSO control and maximum inhibition (by excess anti-TNF biologic, or NF-κB inhibitor–TPCA-1 [Tocris #2559, R&D Systems]). The IC_50_ was determined as the point of inflexion between the fitted minimum and maximum of the compound dose response curve, calculated using 4PL fitted curve. Mean IC_50_s are reported as a mean of at least two experiments. Dose response curves for TNF and the anti-human TNFR1 agonist antibody are shown in Supplementary Fig. 9.

### TNF L929 cytotoxicity assay

TNF (human or mouse) kills L929 cells which have been compromised by incubation with a sub-lethal concentration of actinomycin D, and this effect is inhibited with TNF biologics^18^. Compounds were titrated in DMSO, and then pre-incubated with human or mouse TNF for 1 h. L929 cells (ECACC, #85011425) in media containing actinomycin D (2 µg/mL) were added to the TNF inhibitor mixture and incubated for 18 h. L929 cytotoxicity was measured by the addition of CellTitreGlo (Promega). Luminescence was measured after an incubation of at least 20 min in the dark. Percentage inhibition was calculated between unstimulated wells (maximum signal), and wells with TNF, DMSO and actinomycin D (minimum signal). The IC_50_ was calculated as the point of inflexion for a 4PL fitted curve. Mean IC_50_s are reported as a mean of at least two experiments. Cells were described as mycoplasma-free when purchased and were tested using Mycoalert (Lonza) after each batch of assay vial preparation.

### Mouse or human TNF induced neutrophil recruitment in vivo

All in vivo studies were reviewed by an internal Ethical Review Body (ERB) and conducted in accordance with the Animals (Scientific Procedures) Act 1986, EU Directive 2010/63/EU. Adult male Balb/c mice (aged 6–8 weeks, human: PBS *n* = 4; vehicle *n* = 8; UCB-**9260** 10, 30 and 300 mg/kg *n* = 6 and 100 mg/kg *n* = 8; CDP571 *n* = 5 mice and mouse: PBS *n* = 3; vehicle *n* = 8; UCB-**9260** 10 mg/kg *n* = 7, 30 mg/kg *n* = 6, 100 and 300 mg/kg *n* = 8; CDP571 *n* = 5 mice, from Charles River UK) were dosed orally with vehicle (1% methylcellulose, 400 cps) or compound, 30 min prior to intraperitoneal administration of mouse (0.05 µg/kg) or human (0.03 or 1 µg/kg) TNF diluted in PBS. Anti-TNFα biologics, CDP571^21^ (10 mg/kg) and etanercept (30 mg/kg), diluted in PBS, were dosed intravenously prior to human and mouse TNF challenge. Two hours after the TNF challenge, mice were sacrificed and the peritoneal cavity lavaged with 3 mL of FACS buffer. An aliquot (0.3 mL) of lavage fluid was used to assess the number CD45^+^GR1^+^ cells by flow cytometry. Briefly, the cells were treated with ACK lysis buffer, washed and suspended in FACS buffer prior to staining with FITC anti-mouse CD45 and PE anti-mouse GR1. Data are shown as mean ± standard error of the number of CD45^+^GR-1^+^ cells. Statistical significance (*p < 0.05, **p < 0.01, ****p < 0.0001) was determined using one-way ANOVA with Dunnett’s multiple comparison test.

### Collagen antibody induced arthritis model

Adult male Balb/c mice (aged 6–8 weeks, vehicle *n* = 8; UCB-**9260** *n* = 8 and AB501 *n* = 6 mice, from Charles River UK) received 4 mg of anti-collagen type II antibody cocktail intraperitoneally, which was followed 24 h later with 25 µg of lipopolysaccharide (LPS) intraperitoneally. Dosing with vehicle (30% cyclodextrin po bid) or UCB-**9260** (150 mg/kg po bid) was initiated 6 h post-LPS administration. Ab501 (in-house anti-mouse TNF antibody, 100 m/kg) was administered intravenously 6 h post-LPS administration followed by three further doses administered subcutaneously at 48-h intervals. Animals were scored for signs of arthritis and weighed daily. The following scoring system was used for signs of arthritis: 0-no arthritis, 1-wrist/ankle affected, 2-wrist/ankle and pad affected and 3-wrist/ankle, pad, and digits affected. Data shown as the mean ± standard error of arthritis clinical score for the treatment group. Statistical significance (*p < 0.05, **p < 0.01) was determined using one-way ANOVA with Dunnett’s multiple comparison test.

All in vivo studies were reviewed by an internal ethical review body and conducted in accordance with the Animals (Scientific Procedures) Act 1986, EU Directive 2010/63/EU. Animals were kept under a light/dark cycle of 12/12 h and had access to food and water ad libitum.

### Zymosan induced neutrophil recruitment in vivo

Adult male Balb/c mice (aged 6–8 weeks, PBS *n* = 5; vehicle *n* = 8; UCB-**9260** 10, 30, 100 and 300 mg/kg *n* = 8; Ab501 *n* = 3 mice, from Charles River UK) were dosed intravenously with 30 mg/kg of Ab501 (in-house anti-mouse TNF antibody) diluted in PBS 1 h prior to zymosan challenge. Vehicle (30% cyclodextrin) or UCB-**9260** at 10, 30, 100 and 300 mg/kg was dosed orally at the time of zymosan challenge. Zymosan (R&D Systems) was administered by intraperitoneal injection at a concentration of 1 µg per mouse in PBS (100 µL). One hour post oral administration of UCB-**9260**, mice were bled via the tail to analyse exposure levels of the compound. Four hours after the zymosan challenge, mice were sacrificed and the peritoneal cavity lavaged with 3 mL of FACS buffer. An aliquot (0.3 mL) of lavage fluid was used to assess the number of CD45^+^GR1^+^ cells by flow cytometry. Briefly, the cells were treated with ACK lysis buffer, washed and suspended in FACS buffer prior to staining with FITC anti-mouse CD45 and PE anti-mouse GR1 (BD Biosciences). Data are shown as mean ± standard error of the number of CD45^+^GR-1^+^ cells. Statistical significance (*p < 0.05, **p < 0.01, ***p < 0.001 and ****p < 0.0001) was determined using one-way ANOVA with Dunnett’s multiple comparison test.

### Reporting summary

Further information on research design is available in the Nature Research Reporting Summary linked to this article.

## Supplementary information

Supplementary Information

Peer Review File

Description of Additional Supplementary Files

Supplementary Movie 1

Reporting Summary

## Data Availability

The data supporting this study are available from the corresponding author upon resoanable request. The structural data have been deposited with the Protein Data Bank under accession codes 6OOY for UCB-6876, 6OOZ for UCB-5307, and 6OP0 for UCB-9260. NMR spectral data are available for UCB-**5307** (Supplementary Figs. 13, 14) and for UCB-**9260** (Supplementary Figs. 16, 17). High resolution mass spectrometry (HRMS) is also available for UCB-**5307** (Supplementary Fig. 15) and UCB-**9260** (Supplementary Fig. 18). The source data underlying Figs. 4 and 6, Supplementary Figs. 6–9 and 12, and Supplementary Tables 4–6 are provided in the source data files.

## Source Data

Source Data
