# Supplementary material for: Small molecules that inhibit TNF signalling by stabilising an asymmetric form of the trimer
Source: Nat Commun. 2019 Dec 19;10:5795. doi: 10.1038/s41467-019-13616-1 (PMC6923382; doi:10.1038/s41467-019-13616-1)
Supplement: Supplementary file 1 — Supplementary Information [file 41467_2019_13616_MOESM1_ESM.pdf]

Small molecules that inhibit TNF signalling by stabilising an asymmetric form of the trimer

James O'Connell*[1], John Porter[1], Boris Kroeplien[1], Tim Norman[1], Stephen Rapecki[1], Rachel Davis[1], David McMillan[1], Tracy Arakaki[2], Alex Burgin[3], David Fox III[4], Tom Ceska[1], Fabien Lecomte[1], Alison Maloney[1], Alex Vugler[1], Bruce Carrington[1], Ben Cossins[1], Tim Bourne[1] & Alastair Lawson[1]

[1]UCB Pharma, Slough, SL1 3WE, UK, [2]Covance Inc, Princeton, NJ, USA, [3]Broad Institute of Harvard and MIT, Cambridge, MA, USA, [4]UCB Pharma, Bainbridge Island, WA, 98110, USA

[*]Corresponding author: james.oconnell@ucb.com
UCB Pharma 208 Bath Road, Slough, Berkshire, SL1 3WE

Supplementary Note 1: Experimental and analytical details for synthetic analogues

Materials and reaction conditions.

All solvents and reagents were used as received from commercial suppliers, unless noted otherwise. All reactions involving air- or moisture-sensitive reagents were performed under a nitrogen atmosphere using dried solvents and glassware. UCB-**6876**: 1-[(2,5-dimethylphenyl)methyl]-*1H*-Benzimidazole-2-methanol (CAS 637324-45-9); UCB-**6786**: 1-[(2-methylphenyl)methyl]-*1H*-Benzimidazole-2-methanol (CAS 537018-21-6) were purchased at ASINEX.

Nomenclature

Compounds were named with the aid of ACD/Name Batch (Network) version 11.01 and/or Accelrys Draw 4.0 (IUPAC).

Abbreviations

Br:	Broad
DCM:	Dichloromethane

DMF:	N,N-Dimethylformamide
DMSO:	Dimethylsulfoxide
EtOAc:	Ethyl acetate
ES+:	Electrospray positive ionisation
h:	Hour
H:	Hertz
HPLC:	High performance liquid chromatography
HRMS:	High resolution mass spectroscopy
LCMS:	Liquid Chromatography Mass Spectrometry
M:	Mass
Min:	Minute
MeOH:	Methanol
$MgSO_4$:	Magnesium sulfate
Na_2SO_4:	Sodium sulfate
Pos:	Positive
Neg:	Negative
r.t.:	Room temperature
RT:	Retention time
THF:	Tetrahydrofuran

Analysis by NMR

1H NMR spectra were recorded on a Bruker Avance III HD 500, 400 or 300 MHz spectrometer. 13C NMR spectra were recorded on a Bruker Avance III HD 126 or 101 MHz spectrometer. The chemical shifts (δ) reported are given in parts per million (ppm) and the coupling constants (J) are in Hertz (Hz). The spin multiplicities are reported as s = singlet, d = doublet, t = triplet, q = quartet, dd = doublet of doublet, ddd = doublet of doublet of doublet, dt = doublet of triplet, td = triplet of doublet, and m = multiplet. Spectra were processed using MestReNova 10.0.

Analysis by LC-MS

LC-MS was performed on an Agilent 1200-6120 LC-MS system. LC was performed using HPLC methods 1, 2 or 3 described below and eluted compounds were analysed with an Agilent 6120 mass quadrupole, using Electrospray Ionisation (ESI):

Capillary Voltage 4000 V

Quad Temperature 100 ºC

Drying Gas Flow Rate10 L/min

Drying Gas Temperature 350 °C

Data were acquired from 120 to 1000 m/z

Automated preparative reverse phase HPLC

HPLC purification was performed on a Gilson system with a Gilson 306 pump, Gilson 215 autoinjector, Gilson 215 Fraction collector and a Gilson 156 UV detector, using HPLC method 4 described below.

HPLC methods

Method 1:

Column: Waters X-Bridge, C18, 2.1 x 20 mm, 2.5 μm column.

Mobile phase A: 10 mM ammonium formate in water + 0.1% formic acid

Mobile phase B: acetonitrile + 5% mobile phase A + 0.1% formic acid

Gradient program (flow rate 1.0 mL/min, column temperature 40ºC):

Time	A%	B%
0.00	95.0	5.0
4.00	5.0	95.0
5.00	5.0	95.0
5.10	95.0	5.0

Method 2:

Column: Waters XSelect (C18, 30 x 2.1 mm, 3.5 μm) valve: 1

Flow Rate: 1 mL/minute

Column Temperature: 35ºC

Eluent A: 0.1% formic acid in acetonitrile

Eluent B: 0.1% formic acid in water

Lin. Gradient: t=0 min 5% A, t=1.6 min 98% A, t=3 min 98% A

Detection: DAD (220-320 nm)

Detection: MSD (ESI pos/neg) mass range: 100-800

Detection: ELSD (PL-ELS 2100): gasflow 1.2 mL/min, gas temp: 70ºC, neb: 50°C

Method 3:

Column: Waters XSelect (C18, 50 x 2.1 mm, 3.5 µm) valve: 2

Flow Rate: 0.8 mL/minute

Column Temperature: 35ºC

Eluent A: 0.1% formic acid in acetonitrile

Eluent B: 0.1% formic acid in water

Lin. Gradient: t=0 min 5% A, t=3.5 min 98% A, t=6 min 98% A

Detection: DAD (220-320 nm)

Detection: MSD (ESI pos/neg) mass range: 100-800

Detection: ELSD (PL-ELS 2100): gasflow 1.2 mL/min, gas temp: 70ºC, neb: 50ºC

Method 4:

Column: Luna C18, 21.2 mm, 5 mm column, pH 2.5.

Mobile phase A: 99.92% water and 0.08% formic acid.

Mobile phase B: 99.92% acetonitrile and 0.08% formic acid.

Gradient program (flow rate 25 mL/min, column temperature ambient): variable gradient.

Analysis by HRMS

All final compounds were analysed by HRMS on an Acquity UPLC - Xevo G2 MS.

Capillary Voltage: 3.0kV

Cone: 30 V

Extraction Cone: 2.0V

Source Temperature: 120 c

Desolvation Temperature: 350 c

Cone Gas (L/h): 0

Desolvation Gas (L/h): 500

Supplementary Note 2: Synthesis of UCB-5307

[1-[(2,5-dimethylphenyl)methyl]benzimidazol-2-yl]-(4-pyridyl)methanol

1-(2,5-Dimethylbenzyl)-1*H*-benzimidazole

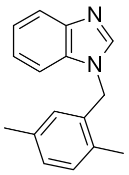

Cesium carbonate (22.0 g, 100.0 mmol) and *n*-butylammonium iodide (12.5 g, 34.0 mmol) were added to a solution of benzimidazole (4.0 g, 34.0 mmol) in DMF (60 mL) at 0ºC. The reaction mixture was stirred for 10 minutes at 0ºC and then 2,5-dimethylbenzyl bromide (6.7 g, 34.0 mmol) was added. The reaction mixture was allowed to warm to room temperature (r.t.) and stirred for 3 h. The mixture was quenched with ice-cold water (50 mL) and extracted with ethyl acetate (3 x 40 mL). The organic layers were dried over anhydrous sodium sulphate and the solvent was removed *in vacuo* yielding 1-(2,5-dimethylbenzyl)-1*H*-benzimidazole (8.0 g, 75%) as an off-white solid. δ_H (300 MHz, d_6-DMSO) 8.23 (s, 1H), 7.68-7.66 (m, 1H), 7.43-7.41 (m, 1H), 7.21-7.19 (m, 2H), 7.10 (d, *J* 7.6 Hz, 1H), 7.01 (d, *J* 7.6 Hz, 1H), 6.67 (s, 1H), 5.45 (s, 2H), 2.25 (s, 3H), 2.14 (s, 3H). LCMS (ES^+) (Method 1) (m/z) [M+H]+ 237.

UCB-5307
[1-(2,5-Dimethylbenzyl)-1*H*-benzimidazol-2-yl](pyridin-4-yl)methanol

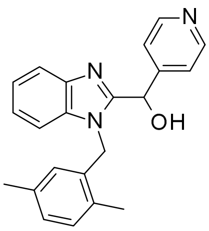

To a solution of 1-(2,5-dimethylbenzyl)-1H-benzimidazole (0.25 g, 1.06 mmol) in THF (10 mL) at -78ºC was added 1.6M *n*-butyllithium (0.79 mL, 1.27 mmol) slowly dropwise and the reaction mixture was stirred for 20 minutes. Isonicotinaldehyde (0.17 g, 1.59 mmol) in THF (1 mL) was added slowly dropwise. After a further 10 minutes the reaction mixture was quenched with water (1 mL) and allowed to warm to r.t. The reaction mixture was poured into ethyl acetate/water. The organic layer was separated, dried ($MgSO_4$) and concentrated *in*

vacuo. The residue was purified by column chromatography (SiO_2, 0-30% MeOH/DCM), yielding [1-(2,5-dimethylbenzyl)-1*H*-benzimidazol-2-yl](pyridin-4-yl)methanol (0.2 g, 55%) as an off-white solid. δ_H ^{1}H NMR (500 MHz, DMSO-d6) δ 8.46 – 8.37 (m, 2H), 7.72 – 7.65 (m, 1H), 7.36 – 7.27 (m, 2H), 7.24 – 7.12 (m, 3H), 7.05 (d, *J* 7.7 Hz, 1H), 6.88 (d, *J* 7.7 Hz, 1H), 6.76 (s, 1H), 6.10 (s, 1H), 5.85 (s, 1H), 5.58 (d, *J* 17.2 Hz, 1H), 5.46 (d, *J* 17.2 Hz, 1H), 2.31 (s, 3H), 1.92 (s, 3H). ^{13}C NMR (126 MHz, DMSO) δ 154.96, 150.20, 149.65, 142.29, 136.19, 135.03, 134.74, 131.95, 130.38, 127.87, 125.69, 123.18, 122.34, 121.61, 119.88, 111.09, 68.28, 45.10, 21.05, 18.77. LCMS (Method 1) (ES^+) (m/z) [M+H]+ 344. HRMS (m/z): [M+H]+ calculated for $C_{22}H_{22}N_3O$, 344.1763; found, 344.1758.
Spectral analysis of UCB-**5307** are shown in Supplementary Figures 13–15.

Supplementary Note 3: Synthesis of UCB-9260

[1-(2,5-Dimethylbenzyl)-6-(1-methyl-1*H*-pyrazol-4-yl)-1*H*-benzimidazol-2-yl](pyridin-4-yl)methanol

5-Bromo-*N*-(2,5-dimethylbenzyl)-2-nitroaniline

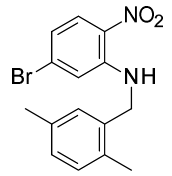

Sodium hydride (60% dispersion in oil, 0.82 g, 20.7 mmol) was added to a stirred solution of 5-bromo-2-nitroaniline (5.0 g, 23.0 mmol) in DMF (50 mL) at 0ºC. 2,5-Dimethyl-benzyl bromide (4.56 g, 23.0 mmol) was added and the reaction mixture was warmed to r.t. and stirred for 5 h. The reaction mixture was quenched with saturated aqueous ammonium chloride solution, extracted with ethyl acetate (3 x 50 mL), washed with water (2 x 30 mL), dried over anhydrous sodium sulfate and concentrated *in vacuo*. The residue was purified by column chromatography (SiO_2, 5% EtOAc/isohexane), yielding 5-bromo-*N*-(2,5-dimethylbenzyl)-2-nitroaniline (4.89 g, 63%) as a yellow solid. δ_H (300 MHz, d_6-DMSO) 8.42 (br s, 1H), 8.01 (d, *J* 8.8 Hz, 1H), 7.12-6.86 (m, 4H), 6.85 (d, *J* 7.2, 1.6 Hz, 1H), 4.54 (d, *J* 5.6 Hz, 2H), 2.28 (s, 3H), 2.21 (s, 3H).

5-Bromo-N^1-(2,5-dimethylbenzyl)benzene-1,2-diamine

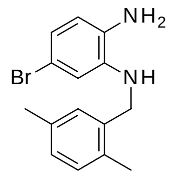

Tin (II) chloride (20.2 g, 89.4 mmol) was added to a stirred solution of 5-bromo-*N*-(2,5-dimethylbenzyl)-2-nitroaniline (10.0 g, 29.8 mmol) in EtOH (200 mL) and the reaction mixture was heated to 80ºC for 5 h. The reaction mixture was then concentrated *in vacuo* and the residue neutralized with saturated aqueous sodium bicarbonate solution and extracted with DCM (3 x 100 mL). The combined organics were washed with water (2 x 50 mL), extracted, dried over anhydrous sodium sulfate and concentrated *in vacuo*. The residue was purified by column chromatography (SiO_2, 5% MeOH/DCM), yielding 5-bromo-N^1-(2,5-dimethylbenzyl)benzene-1,2-diamine (5.4 g, 69%) as a dark brown oil. δ_H (300 MHz, d_6-DMSO) 7.08 (s, 1H), 7.06 (d, *J* 7.6 Hz, 2H), 6.97 (d, *J* 7.6 Hz, 1H), 6.53 (dd, *J* 8.4, 2.0 Hz, 1H), 6.47 (d, *J* 8.0 Hz, 1H), 6.45 (d, *J* 2.0 Hz, 1H), 5.06 (t, *J* 5.4 Hz, 1H), 4.77 (br s, 2H), 4.15 (d, *J* 5.2 Hz, 1H), 2.27 (s, 3H), 2.22 (s, 3H). LCMS (Method 1) (ES^+) (m/z) [M+H]+ 305/307.

6-Bromo-1-(2,5-dimethylbenzyl)-1*H*-benzimidazole

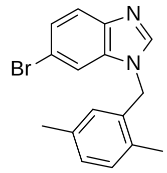

A mixture of 5-bromo-N^1-(2,5-dimethylbenzyl)benzene-1,2-diamine (0.40 g, 1.31 mmol) and formic acid (10 mL) was stirred at r.t. for 18 h. The reaction mixture was concentrated *in vacuo* and the residue partitioned between ethyl acetate and saturated aqueous sodium bicarbonate solution. The organic layer was dried over anhydrous sodium sulphate and concentrated *in vacuo*. The crude residue was purified by column chromatography (SiO_2, 20-75% EtOAc/ isohexane), yielding 6-bromo-1-(2,5-dimethylbenzyl)-1*H*-benzimidazole (0.20 g, 48%) as a white solid. δ_H (300 MHz, d_6-DMSO) 8.24 (s, 1H), 7.74 (d, *J* 1.7 Hz, 1H), 7.64 (d, *J* 8.6 Hz, 1H), 7.34 (dd, *J* 8.6, 1.9 Hz, 1H), 7.12 (d, *J* 7.7 Hz, 1H), 7.02 (d, *J* 7.8 Hz, 1H), 6.61 (s, 1H), 5.47 (s, 2H), 2.24 (s, 3H), 2.15 (s, 3H). LCMS (Method 1) (ES^+) (m/z) [M+H]+ 316/318.

[6-Bromo-1-(2,5-dimethylbenzyl)-1*H*-benzimidazol-2-yl](pyridin-4-yl)methanol

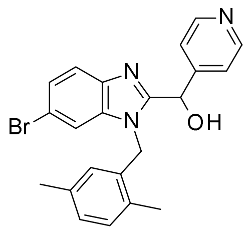

To diisopropylamine (2.8 mL) in THF (10 mL), cooled to 0ºC, was added *n*-butyllithium (12.5 mL, 1.6M in hexanes) and the resulting mixture was stirred at 0ºC for 10 minutes. An aliquot of this freshly prepared lithium diisopropylamide (1.8 mL, 1.62 mmol) was added to a solution of 6-bromo-1-(2,5-dimethylbenzyl)-1*H*-benzimidazole (0.25 g, 0.81 mmol) in THF (5 mL) at -78ºC. The reaction mixture was stirred for 2 h at -78ºC, then pyridine-4-carboxaldehyde (0.15 mL, 1.62 mmol) was added and the reaction mixture was stirred at -78ºC for 10 minutes. The mixture was quenched with saturated aqueous sodium chloride solution and allowed to warm to r.t. The mixture was extracted with ethyl acetate (3 x 40 mL). The organic layers were dried over anhydrous sodium sulphate and concentrated *in vacuo*. The residue was purified by column chromatography (SiO_2, 0-10% MeOH/DCM), yielding [6-bromo-1-(2,5-dimethylbenzyl)-1*H*-benzimidazol-2-yl](pyridin-4-yl)methanol (0.18 g, 51%) as a white solid. δ_H (400 MHz, d6-DMSO) 8.44 – 8.36 (m, 2H), 7.65 (d, *J* 8.6 Hz, 1H), 7.53 (d, *J* 2.0 Hz, 1H), 7.35 (dd, *J* 8.6, 1.8 Hz, 1H), 7.31 – 7.27 (m, 2H), 7.06 (d, *J* 7.7 Hz, 1H), 6.88 (d, 1H), 6.79 (d, *J* 5.5 Hz, 1H), 6.06 (d, *J* 5.4 Hz, 1H), 5.74 (s, 1H), 5.60 (d, *J* 17.3 Hz, 1H), 5.47 (d, *J* 17.3 Hz, 1H), 2.30 (s, 3H), 1.92 (s, 3H). LCMS (Method 1) (ES^+) (m/z) [M+H]+ 423/425.

UCB-9260

[1-(2,5-Dimethylbenzyl)-6-(1-methyl-1*H*-pyrazol-4-yl)-1*H*-benzimidazol-2-yl](pyridin-4-yl)methanol

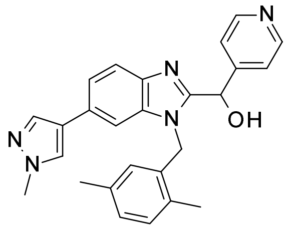

1-Methyl-4-(4,4,5,5-tetramethyl-[1,3,2]dioxaborolan-2-yl)-1*H*-pyrazole (0.064 g, 0.31 mmol), and a 2M aqueous solution of sodium carbonate (1 mL) were added to a solution of [6-bromo-1-(2,5-dimethylbenzyl)-1*H*-benzimidazol-2-yl](pyridin-4-yl)methanol (0.12 g, 0.29 mmol) in 1,4-dioxane:water (4:1, 5 mL) and the reaction mixture was degassed for 10 minutes. [1,1′-Bis(diphenylphosphino)ferrocene]dichloropalladium(II) (0.01 mg, 0.05 mmol) was added and the reaction mixture was degassed for 10 minutes, then heated to 100ºC for 60 minutes in a Biotage microwave reactor. Ethyl acetate was added and the mixture filtered through a Celite pad. The organic layer was separated, dried over anhydrous sodium sulphate, and concentrated *in vacuo*. The residue was purified by preparative HPLC (method 4), yielding [1-(2,5-dimethylbenzyl)-6-(1-methyl-1*H*-pyrazol-4-yl)-1*H*-benzimidazol-2-yl](pyridin-4-yl)methanol as a white solid (0.076 g, 62%). δ_H (400 MHz, d6-DMSO). 8.44 – 8.35 (m, 2H), 8.04 (s, 1H), 7.77 (d, *J* 0.8 Hz, 1H), 7.64 (d, *J* 8.3 Hz, 1H), 7.48 – 7.40 (m, 2H), 7.32 – 7.25 (m, 2H), 7.07 (d, *J* 7.6 Hz, 1H), 6.88 (d, *J* 7.7 Hz, 1H), 6.73 (broad s, 1H), 6.02 (s, 1H), 5.81 (s, 1H), 5.60 (d, *J* 17.3 Hz, 1H), 5.46 (d, *J* 17.3 Hz, 1H), 3.83 (s, 3H), 2.34 (s, 3H), 1.92 (s, 3H). ^{13}C NMR (101 MHz, DMSO) δ 154.96, 150.13, 149.58, 140.86, 136.95, 136.40, 135.03, 134.93, 131.93, 130.35, 128.11, 128.06, 127.80, 125.39, 122.95, 121.60, 120.36, 120.16, 106.78, 68.17, 44.97, 39.06, 21.09, 18.80. LCMS (Method 1) (ES^+) (m/z) [M+H]+ 424. HRMS (m/z): [M+H]+ calculated for $C_{26}H_{26}N_5O$, 424.2137; found, 424.2139. Spectral analysis of UCB-**9260** are shown in Supplementary Figures 16–18.

Supplementary Note 4: UCB-6786 and UCB-9260 are not colloidal aggregators

Dynamic light scattering (DLS) experiments have been performed with compounds UCB-**6786** and UCB-**9260** at 100 µM and 10 µM respectively, as requested. A buffer baseline control and positive control (miconazole at 100 µM) were included. The radius data (n=5) are plotted below, and clearly show no evidence of colloidal aggregation for either compound in the absence of detergent.

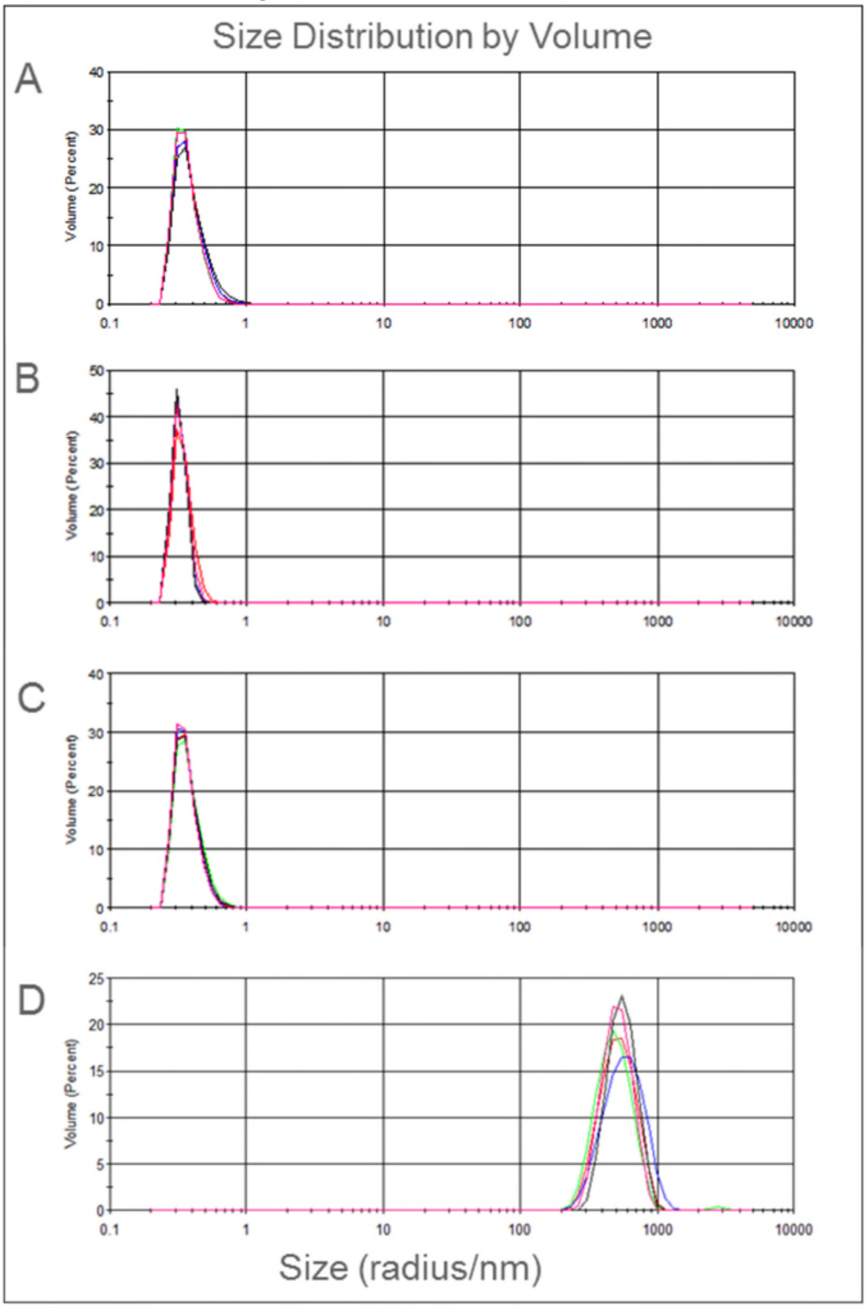

A=Buffer; B=100 µM UCB-**6786**; C=10 µM UCB-**9260**; D=100 µM Miconazole (positive control).

DLS data (Malvern Nano ZS). Each sample (150 µl) was measured 5 times in a low volume disposable sizing cuvette at 25°C using 173° non-invasive backscatter (NIBS).

Experiments to show any effect of addition of non-ionic detergent were performed on a Biacore, with human TNF on the solid phase and compounds in solution phase in the presence and absence of 0.01% v/v Triton X-100 and 0.025% v/v Tween-80.

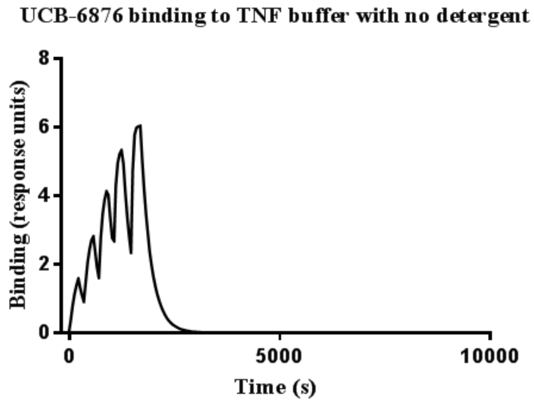

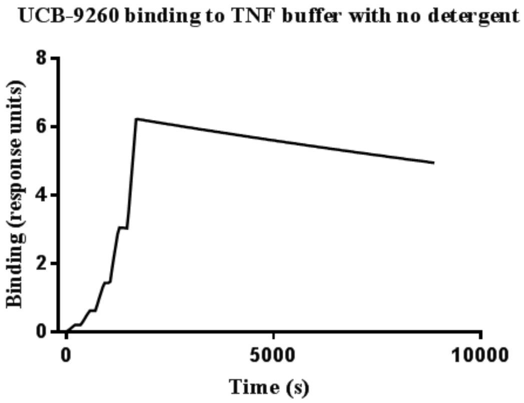

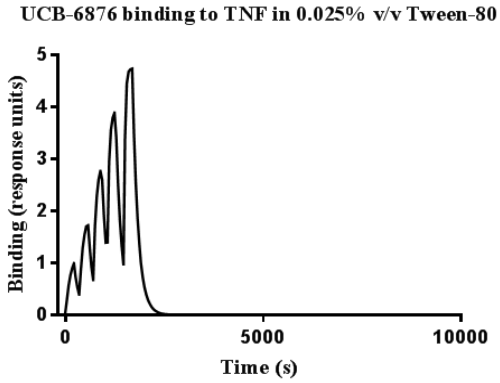

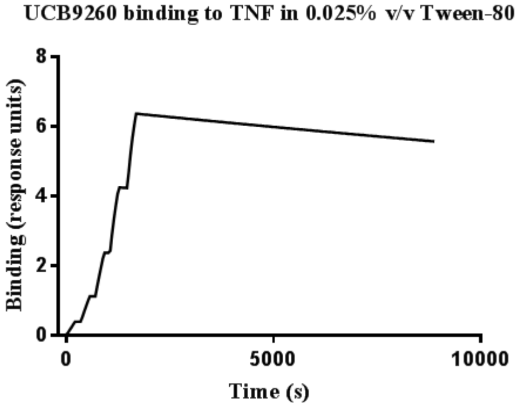

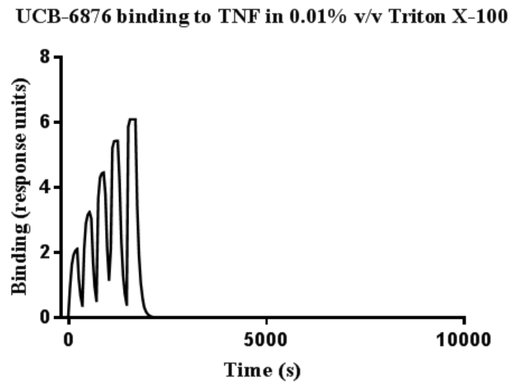

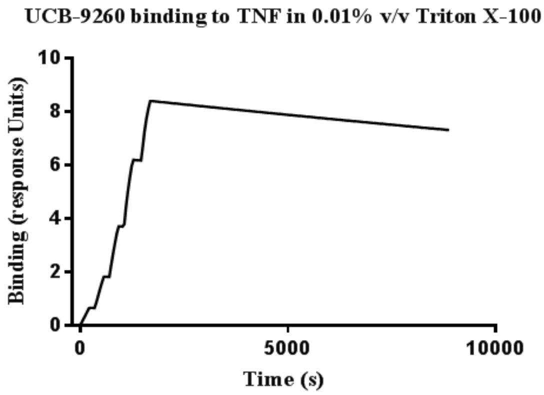

These traces indicate that the addition of the specified detergents does not alter the binding of compounds UCB-**6876** and UCB-**9260** to human TNF.

Methods:
Three Biacore T200s were treated with sodium hypochlorite to remove any traces of detergent. Each machine was equilibrated in either HBS with no detergent (control), HBS: Tween-P80 (0.025% v/v) or HBS: Triton X-100 (0.01% v/v). Human TNF was tethered to ~ 2000 RU before equilibration in the equivalent control or detergent buffers plus 1% DMSO. Compounds were doubly diluted and run at 1.875, 3.75, 7.5, 15, 30 µM (UCB-**6786**) and from 0.625, 1.25, 2.5, 5, 10 µM (UCB-**9260**) by single cycle kinetics. The referenced data were fitted to a BIAevaluation T200 1:1 algorithm and the fitted data shown.

Supplementary Figures

a

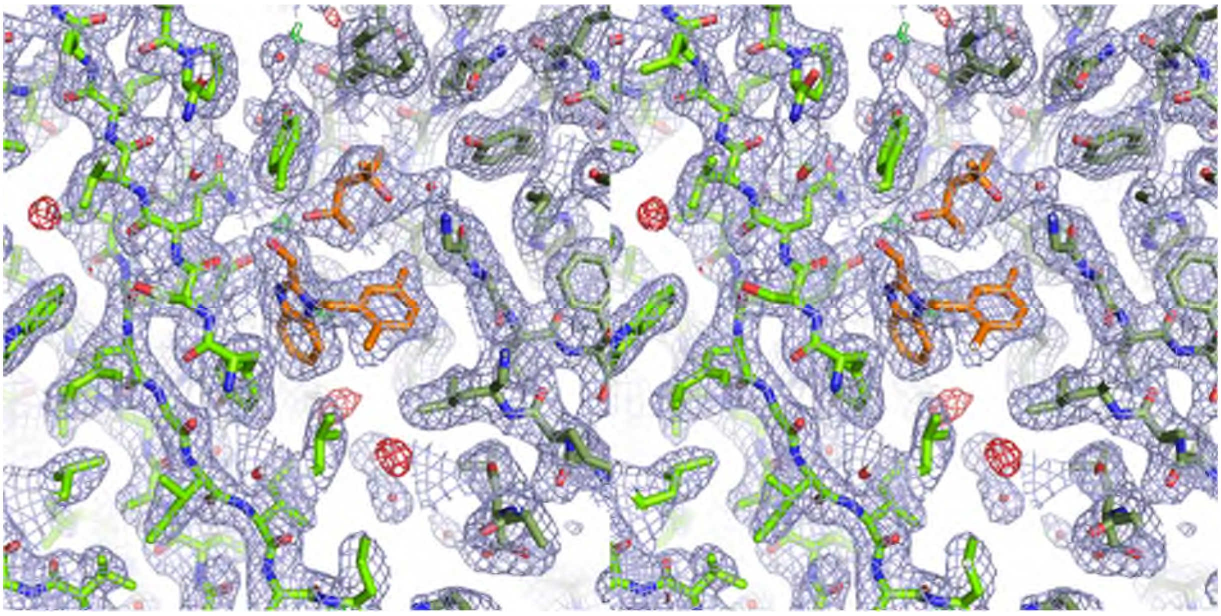

b

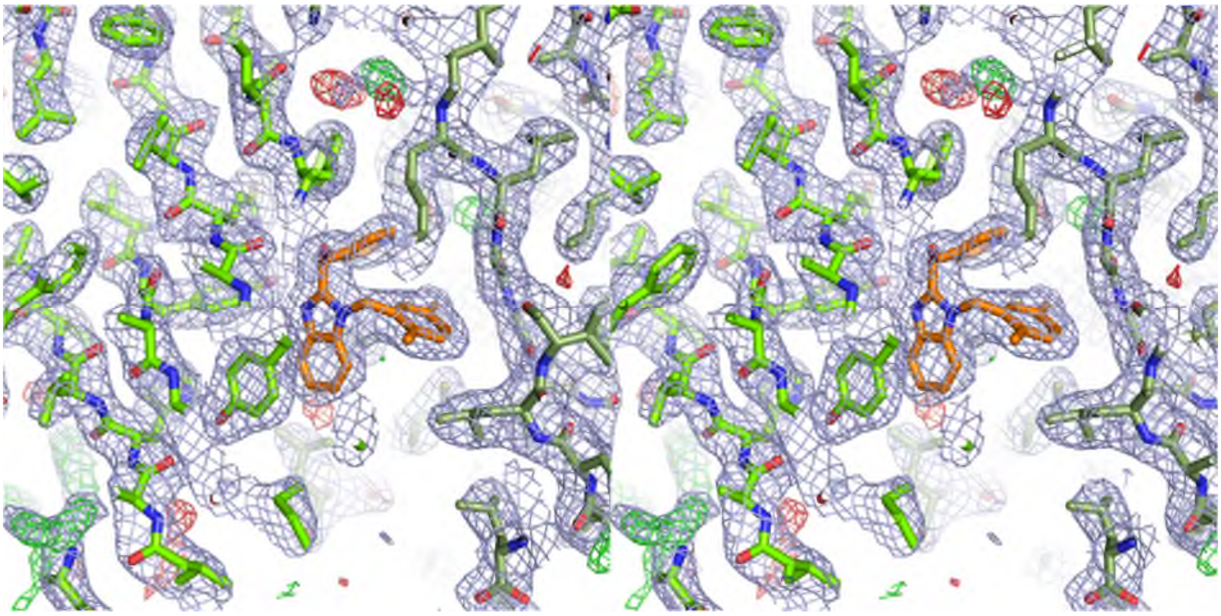

c

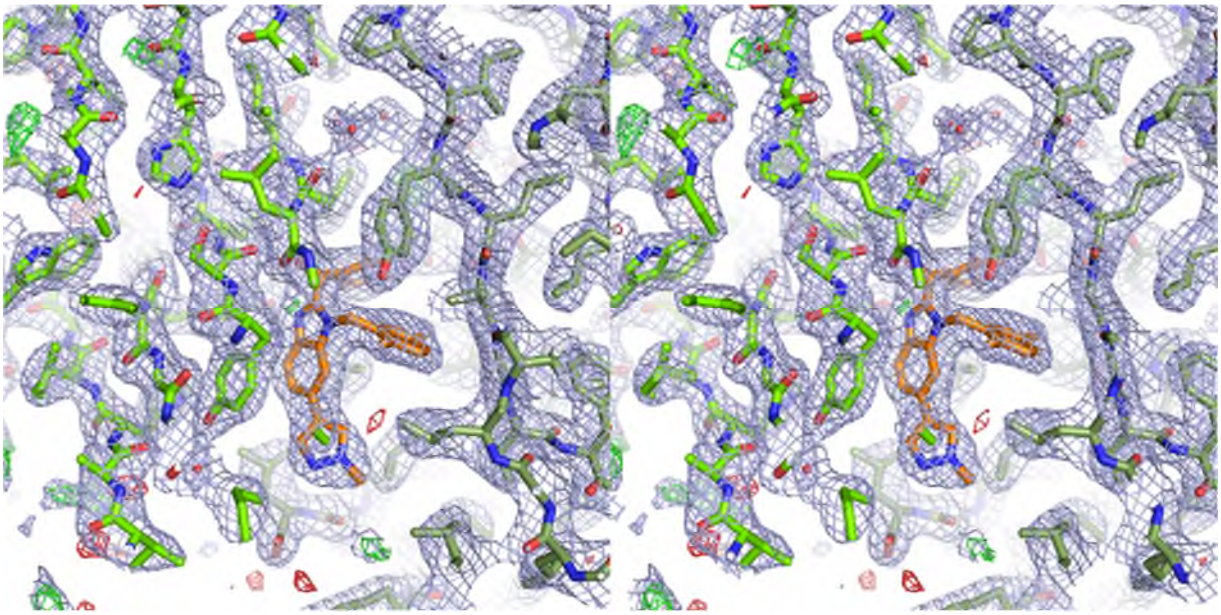

Supplementary Fig. 1: Stereo images of electron density
(a) Human TNF (bound with UCB-**6876** and 2-Methyl-2,4-pentanediol). Trimeric human TNF (green sticks) with UCB-**6876** and 2-Methyl-2,4-pentanediol (orange sticks). 2Fo-Fc maps contoured at 1.0σ (blue mesh), Fo-Fc maps contoured at ±3.0σ (green/red mesh). (b) Human TNF (bound with UCB-**5307**). Trimeric human TNF (green sticks) with UCB-**5307** (orange sticks). 2Fo-Fc maps contoured at 1.0σ (blue mesh), Fo-Fc maps contoured at +/-3.0σ (green/red mesh). (c) Human TNF (bound with UCB-**9260**). Trimeric human TNF (green sticks) with UCB-**9260** (orange sticks). 2Fo-Fc maps contoured at 1.0σ (blue mesh), Fo-Fc maps contoured at +/-3.0σ (green/red mesh).

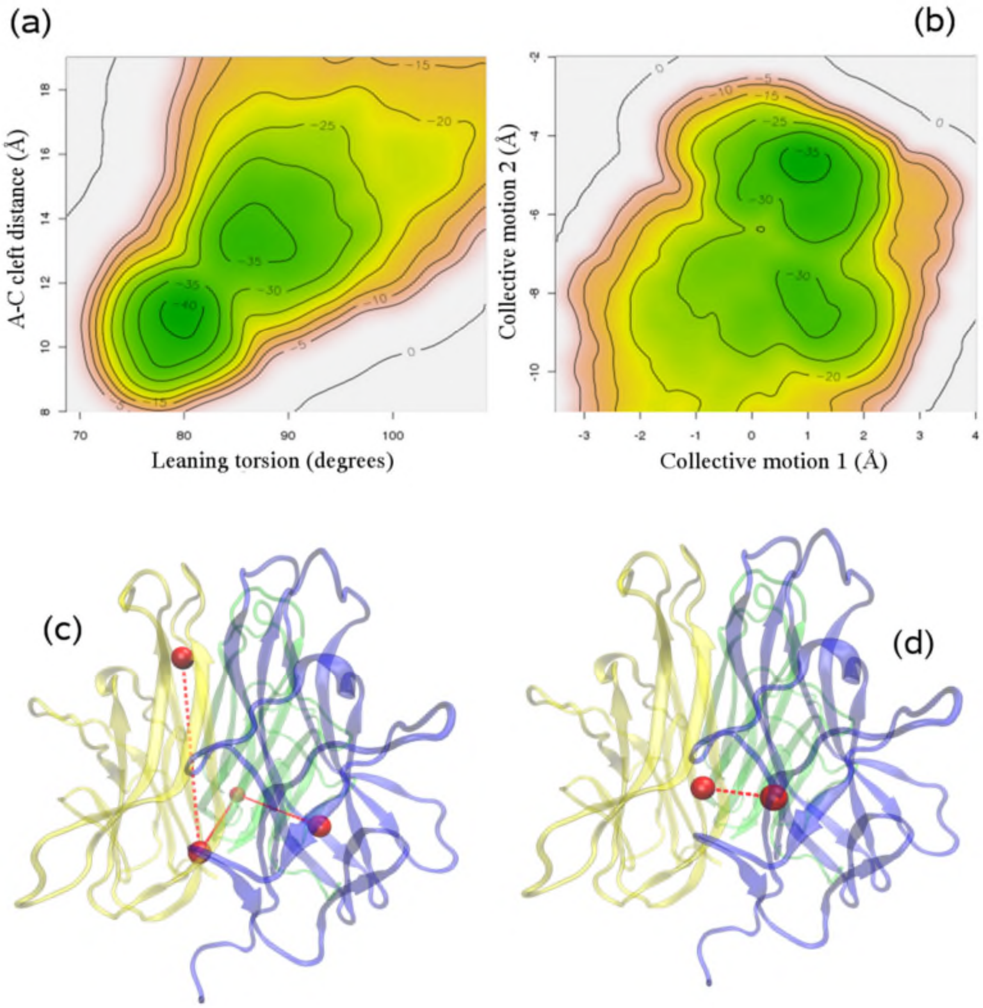

Supplementary Fig. 2: FES diagrams of the opening of TNF without small molecules
(a & b) Free-energy (FE) diagrams with units of kcal.mol^{-1} with green representing the lowest FE regions and white the highest. (b) The axes are the lowest-frequency PCA vector fields produced through PCA analysis of the initial simulations (a). (c) A descriptor of human TNF conformation (CVs highlighted with red spheres; centres of mass of α-carbon atoms and connecting lines) using in the x-axis of (a). (d) The descriptor of human TNF conformation using in the y-axis of (a).

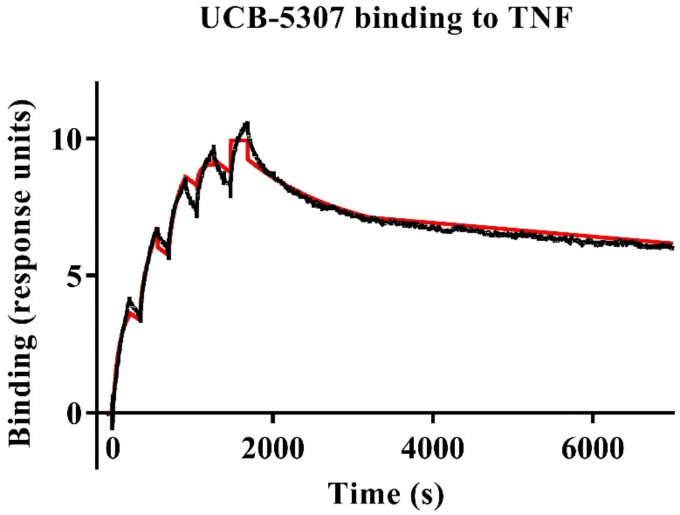

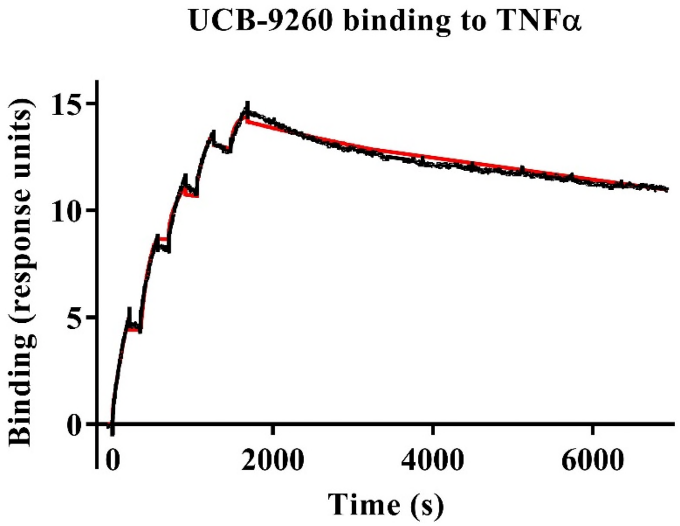

	ka (1/Ms)	kd (1/s)	$T_{1/2}$ (h)	KD nM
UCB-5307	6368+/-20	5.76E-05+/-0.012	3.3	9
UCB-9260	3175+/-7	4.39E-05+/-0.011	4.4	13.8

Supplementary Fig. 3: Biacore of UCB-9260 and UCB-5307 binding to human TNF

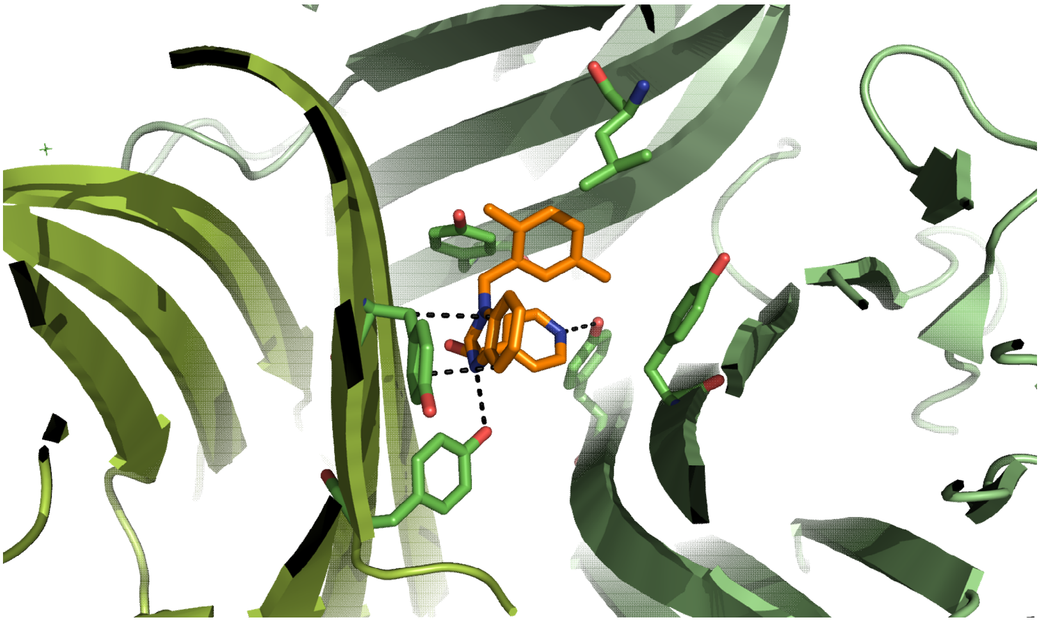

Supplementary Fig. 4: Crystal structure of human TNF with UCB-5307
Detail of UCB-**5307** (racemate) bound within the TNF homotrimer, with key residues involved in binding highlighted (orange and green sticks and red labels).

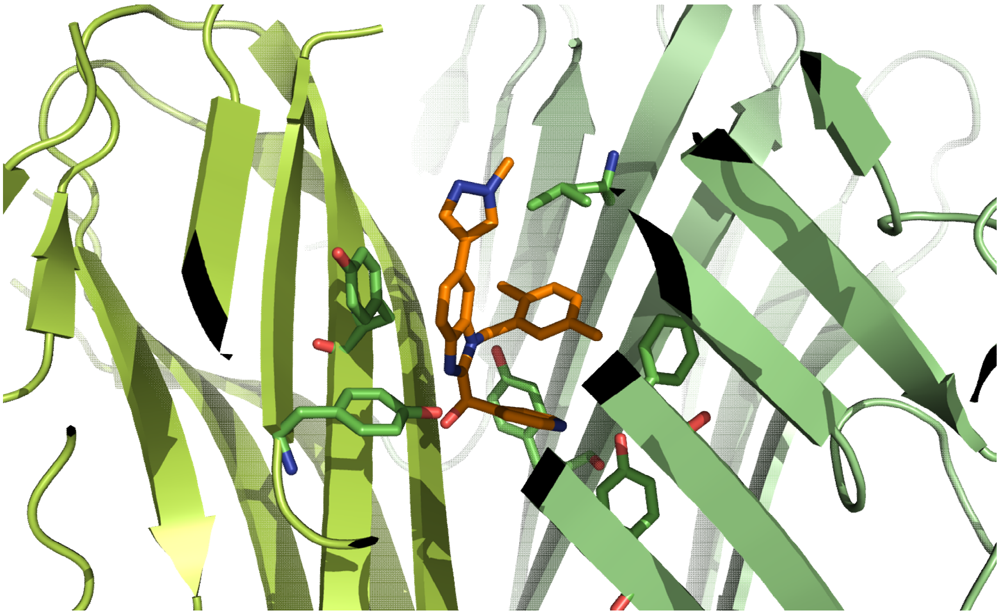

Supplementary Fig. 5: Crystal structure of human TNF with UCB-9260
Detail of UCB-**9260** (racemate) bound within the TNF homotrimer, with key residues involved in binding highlighted (orange and green sticks and red labels) and the three assigned regions of the compound binding pocket (Sites 1-3) highlighted.

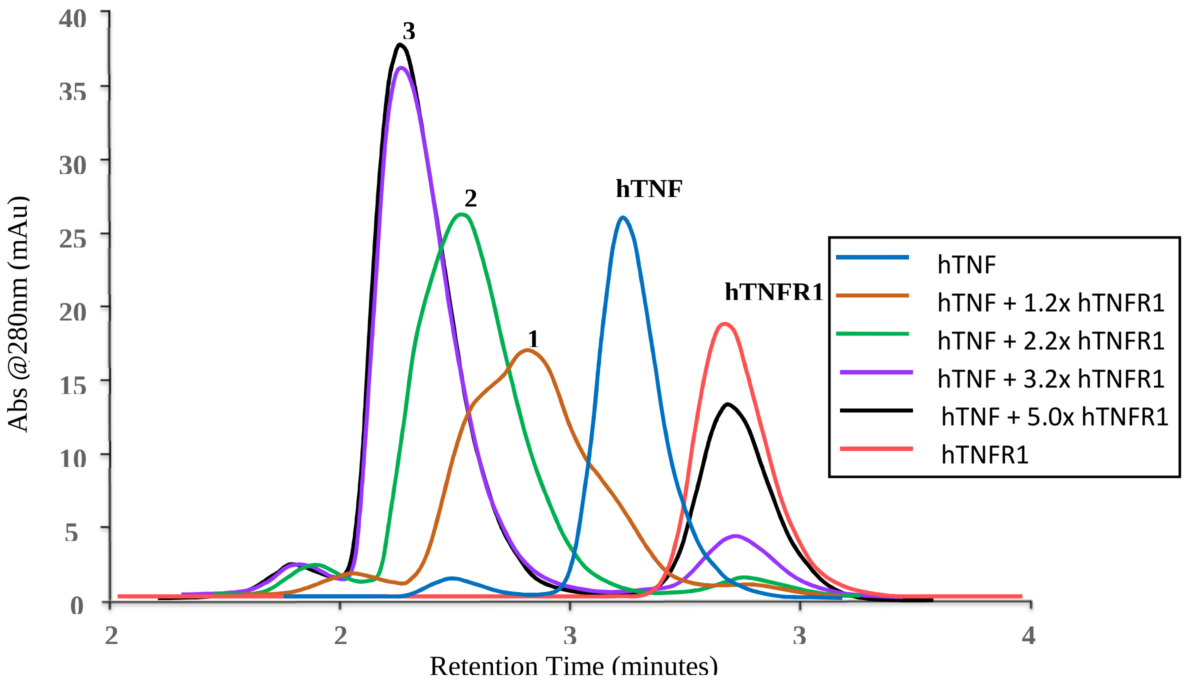

Supplementary Fig. 6: Size exclusion chromatography titration of TNFR1 with TNF Analysis of human TNF (blue trace), human TNFR1 (red trace) and mixtures of the two, over a range of ratios (TNF homotrimer: TNFR1) 1:1.2, 1:2.2, 1:3.2 and 1:5 (brown, green, purple and black traces, respectively). Peaks corresponding to TNF with 1, 2 and 3 receptors bound are numbered. At sub-saturating concentrations of receptor (1.2x & 2.2x, brown and green traces respectively), in addition to the main peaks of 1 and 2 receptors bound, shoulders on these peaks indicate the presence of all possible complex stoichiometries as the mix establishes a state of equilibrium. Source data are provided as a Source Data file.

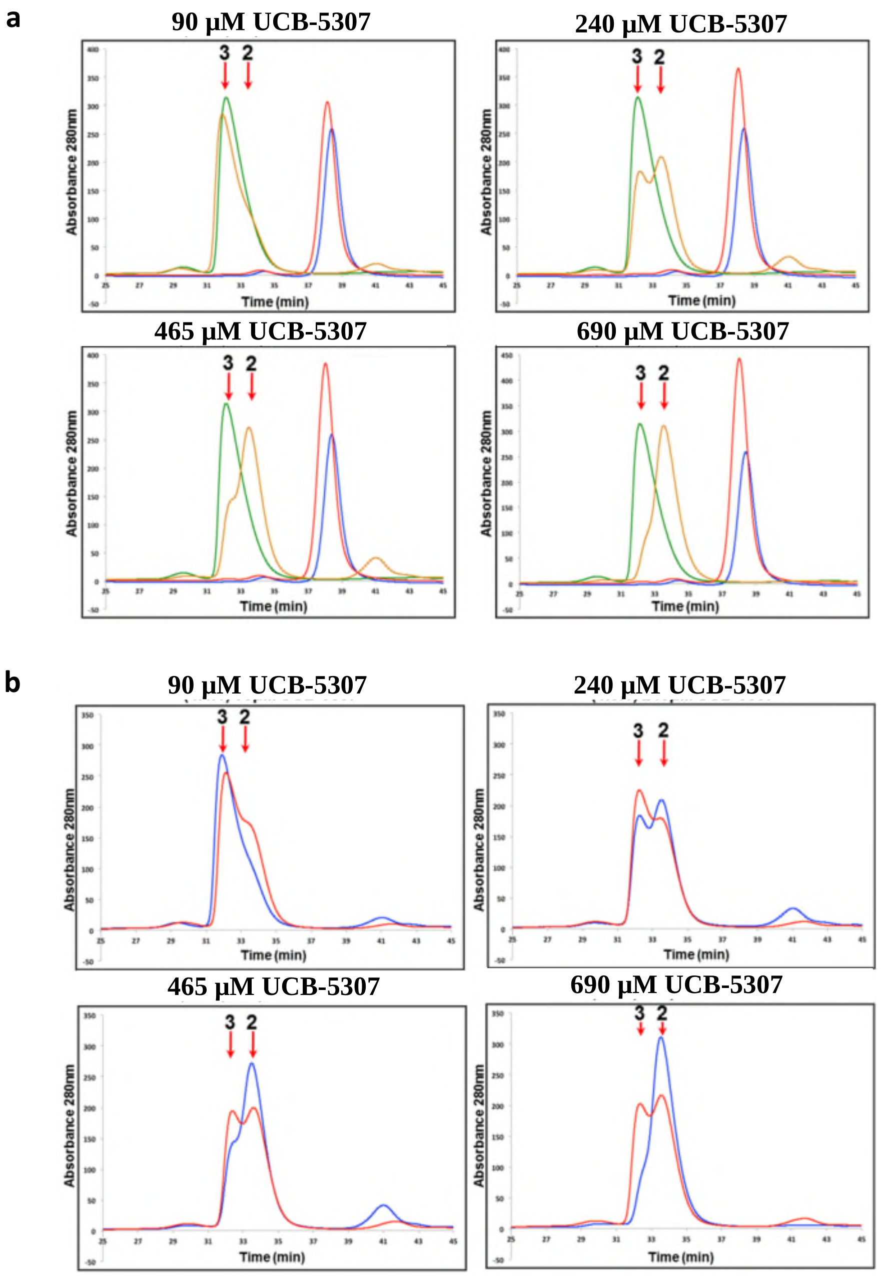

Supplementary Fig. 7: Effect of titrating UCB-5307 on TNF:TNFR1 stoichiometry
(a) Analytical size exclusion chromatography (AnSEC) of human TNF alone (blue trace), human TNF + UCB-**5307** (red trace), human TNF + 3.2 fold excess human TNFR1 (green trace) and human TNF + UCB-**5307** + 3.2 fold excess human TNFR1 (brown trace) at four compound concentrations (expected migration position of human TNF with 2 and 3 receptors bound are indicated by arrows). **(b)** AnSEC comparing effect of four compound concentrations on preformed human TNF/human TNFR1 complex (3.2 fold excess human TNFR1) (red trace) vs human TNF preloaded with UCB-**5307** followed by addition of 3.2 fold excess human TNFR1 (blue trace). Source data are provided as a Source Data file.

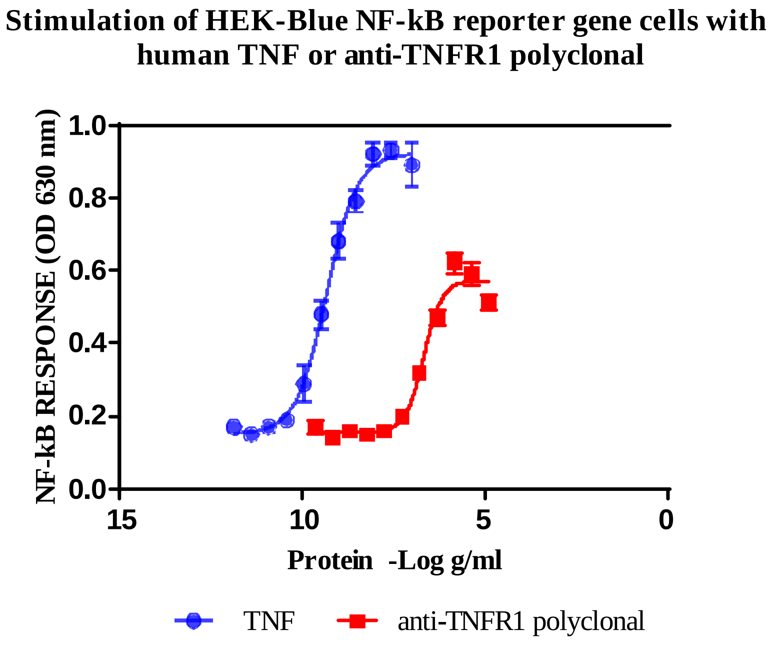

Supplementary Fig. 8: Comparison of TNF and an agonistic antibody to TNFR1
Titration of TNF (VCID: 2043 Beryllium) and anti-TNFR1 polyclonal (R&D Systems. #AF 225) in the TNF stimulated HEK-293 reporter gene assay showing that maximal activation of the NF-kB pathway by the agonist antibody is reduced compared to TNF. Data displayed are mean and SD of quadruplicate from the same dilution series. Source data are provided as a Source Data file.

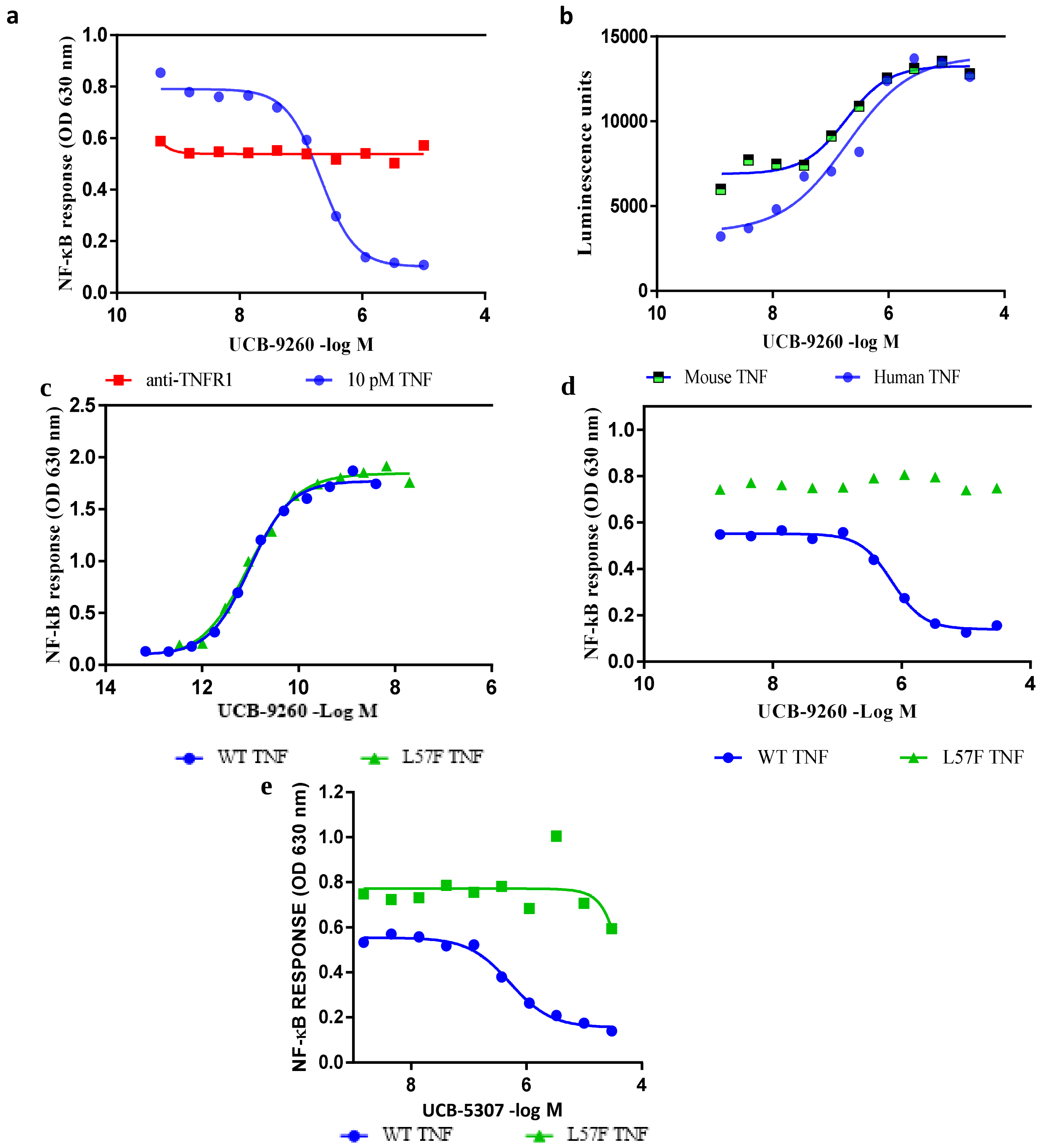

Supplementary Fig. 9: Activity of UCB-9260 and -5307 in cell assays
(a) Untransformed data for Figure 5b showing inhibition of human TNF by UCB-**9260** in the HEK NFκB reporter gene assay but no inhibition (up to 10 µM) of NFκB stimulated by an agonistic receptor antibody. (b) Untransformed data for Fig. 5c showing inhibition of mouse and human TNF by UCB-**9260** in the mouse cell L929 assay. In this assay reduction is luminescence is due to cell killing by TNF and inhibition causes increased luminescence. (c) Titration of wild-type human TNF (WT TNF) and TNF with a single mutation (L57F TNF) showing that they have similar activities (NFκB response) in a HEK reporter gene assay. HEK NFκB reporter gene assay showing that (d) UCB-**9260** and (e) UCB-**5307** inhibited 10 pM wild-type TNF but did not inhibit 10 pM L57F TNF. Source data are provided as a Source Data file.

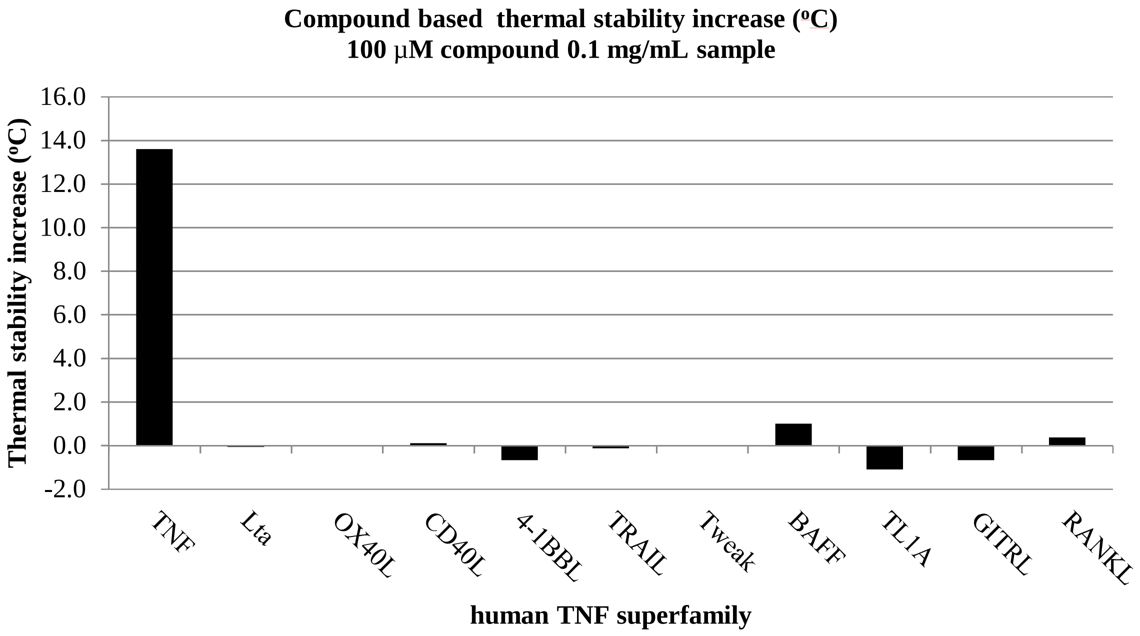

Supplementary Fig. 10: UCB-9260 binding to human TNF superfamily
This shows negligible effect of UCB-**9260** on a number of TNF superfamily members compared to human TNF. Human proteins were provided through a collaboration with Beryllium. Data from a single experiment.

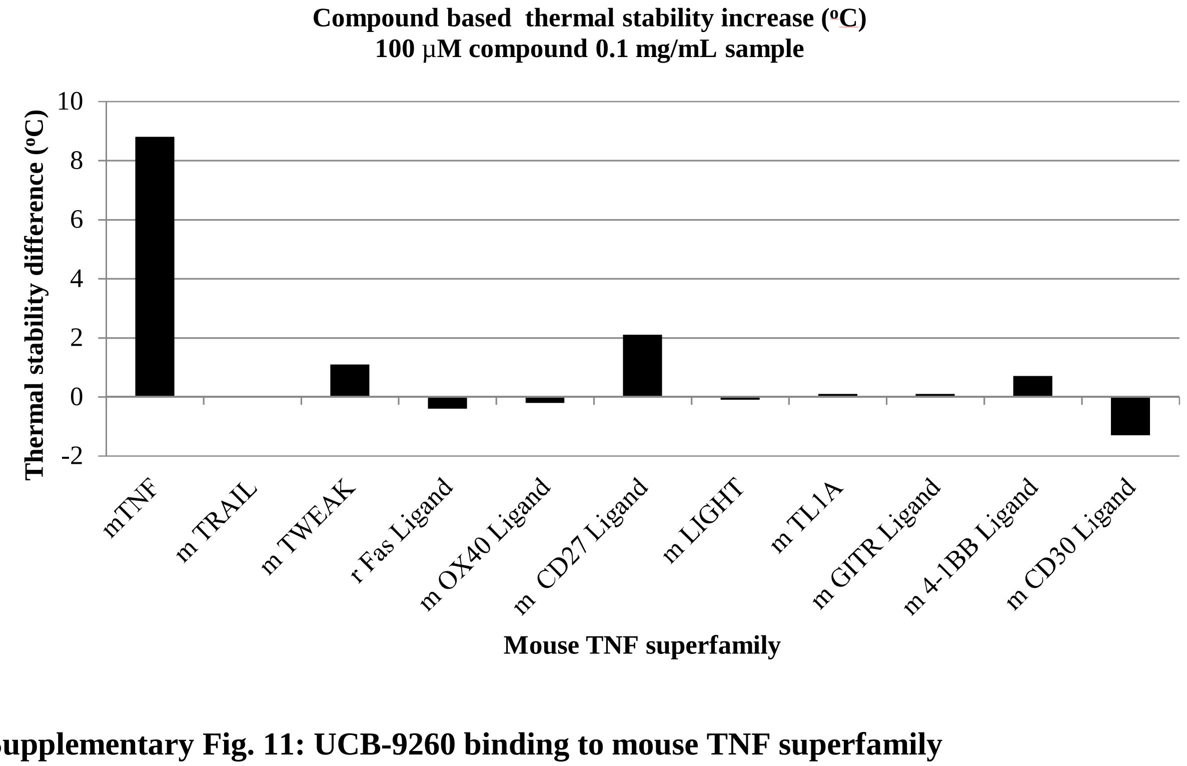

Supplementary Fig. 11: UCB-9260 binding to mouse TNF superfamily
This shows negligible effect of UCB-**9260** on a number of TNF superfamily members compared to mouse TNF. Mouse proteins were purchased from R&D Systems. (Note: No Tm for Lymphotoxin (LTa) was detected in this assay making a change in Tm unmeasurable). Data from a single experiment.

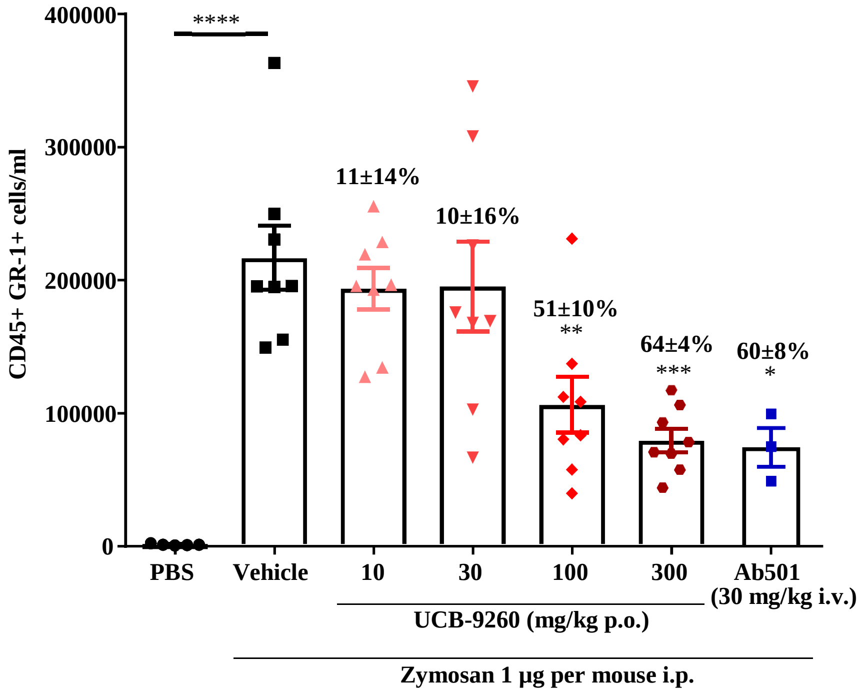

Supplementary Fig. 12: UCB-9260 inhibits zymosan-induced neutrophil recruitment
Zymosan-administered i.p. resulted in a statistically significant recruitment of neutrophils to the peritoneal cavity at 4 hours post challenge. UCB-**9260** (10–300 mg/kg p.o. at the time of challenge) dose-dependently inhibits zymosan-induced neutrophil recruitment to the peritoneal compartment in mice (n=5 PBS, n=8 vehicle, 10, 30, 100 & 300mg/kg UCB-**9260** and n=3 Ab501 mice/group). Table shows exposure of UCB-**9260** 1 hour post dose. Free unbound exposure in excess of the *in vitro* IC_{50} (95 nM) was required for a statistically significant inhibition. Mean data ± s.e. is shown. One-way ANOVA with Dunnett's multiple comparisons post-test *p<0.05, **p<0.01, *** p<0.001 and ****p<0.0001. Source data are provided as a Source Data file.

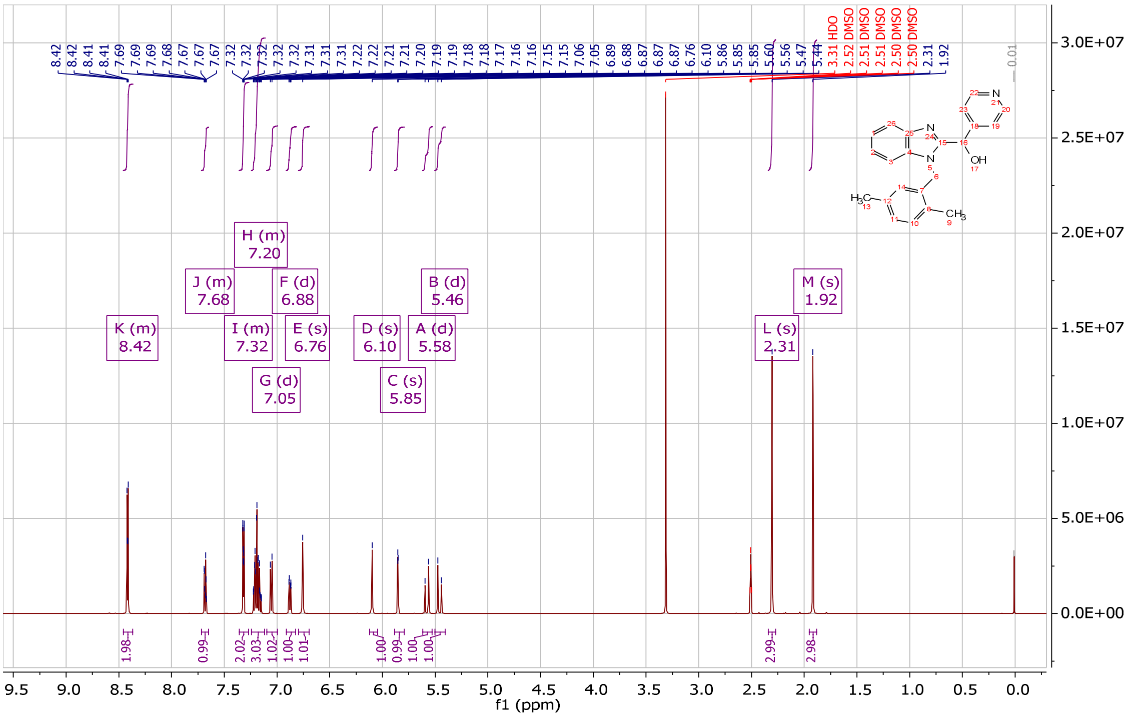

Supplementary Fig. 13: 1H NMR spectrum of UCB-5307

The spectrum was recorded on a Bruker Avance III HD 500 MHz spectrometer.

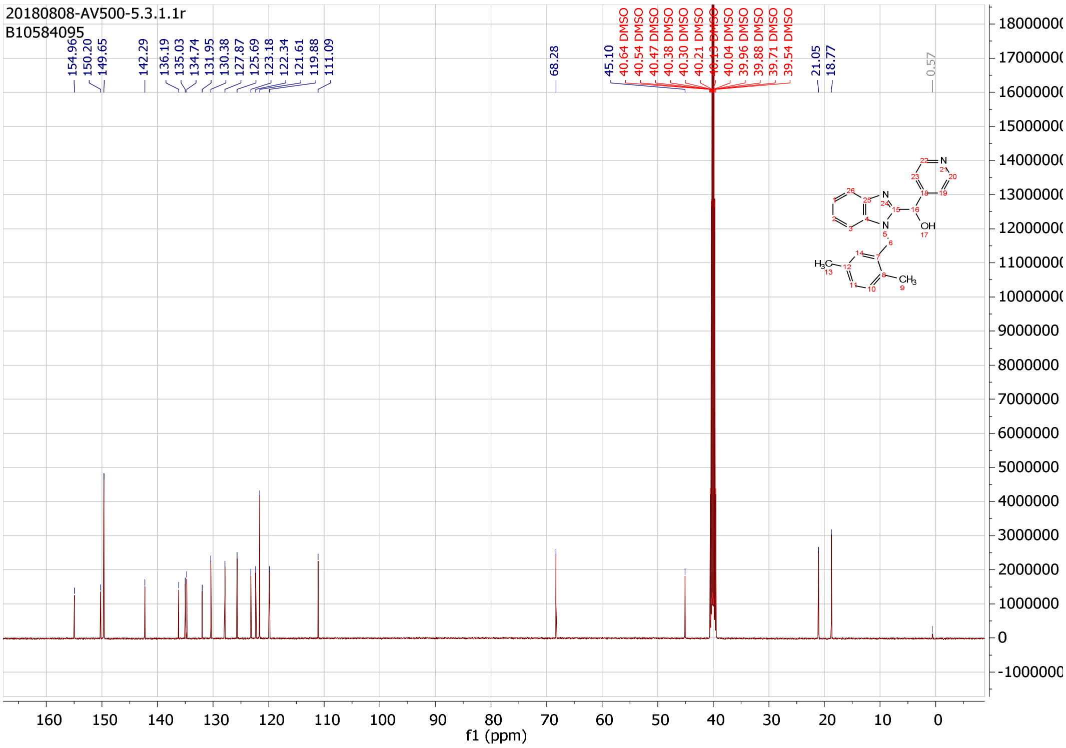

Supplementary Fig. 14: 13C NMR spectrum of UCB-5307 (126 MHz)

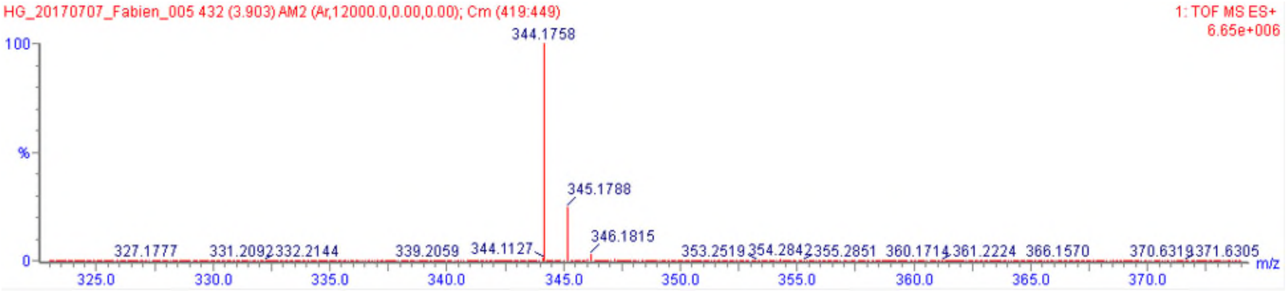

Supplementary Fig. 15: HRMS spectrum of UCB-5307

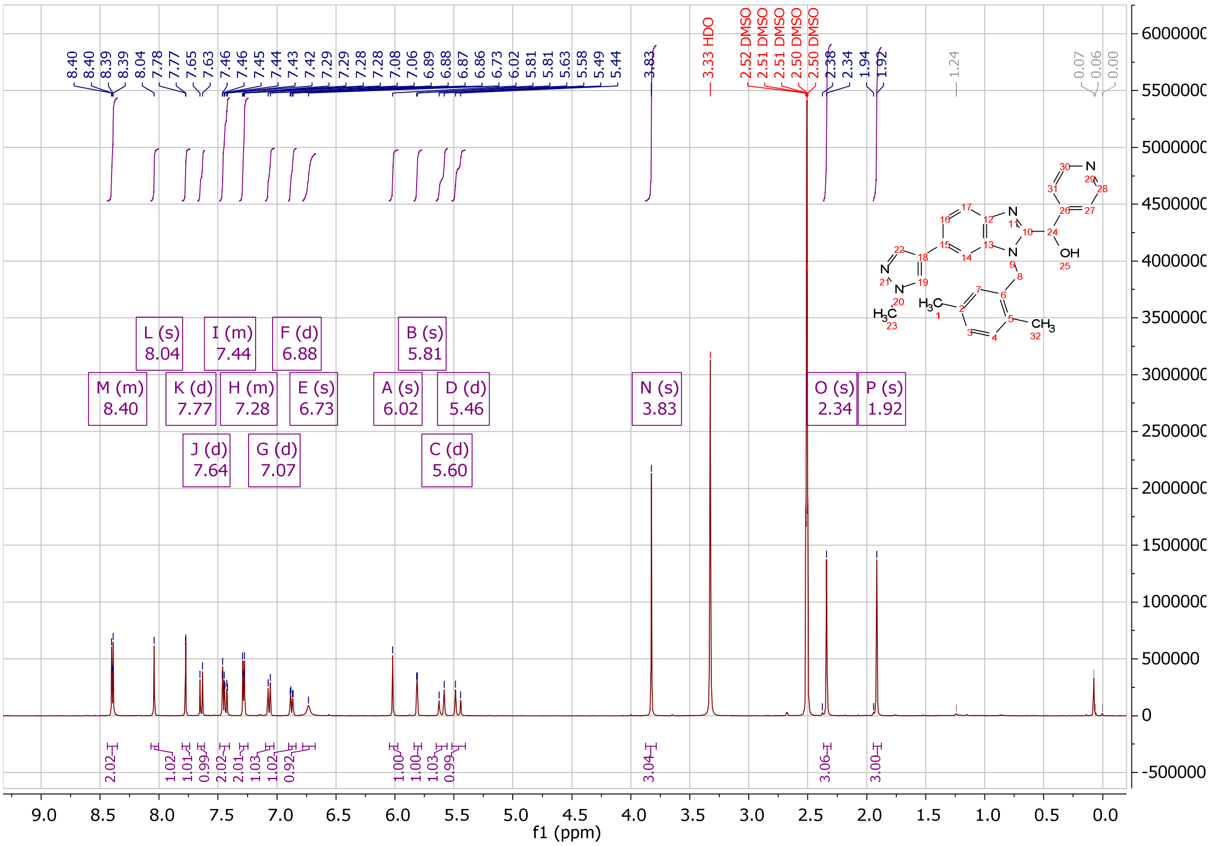

Supplementary Fig. 16: 1H NMR spectrum of UCB-9260

The spectrum was recorded on a Bruker Avance III HD 400 MHz spectrometer.

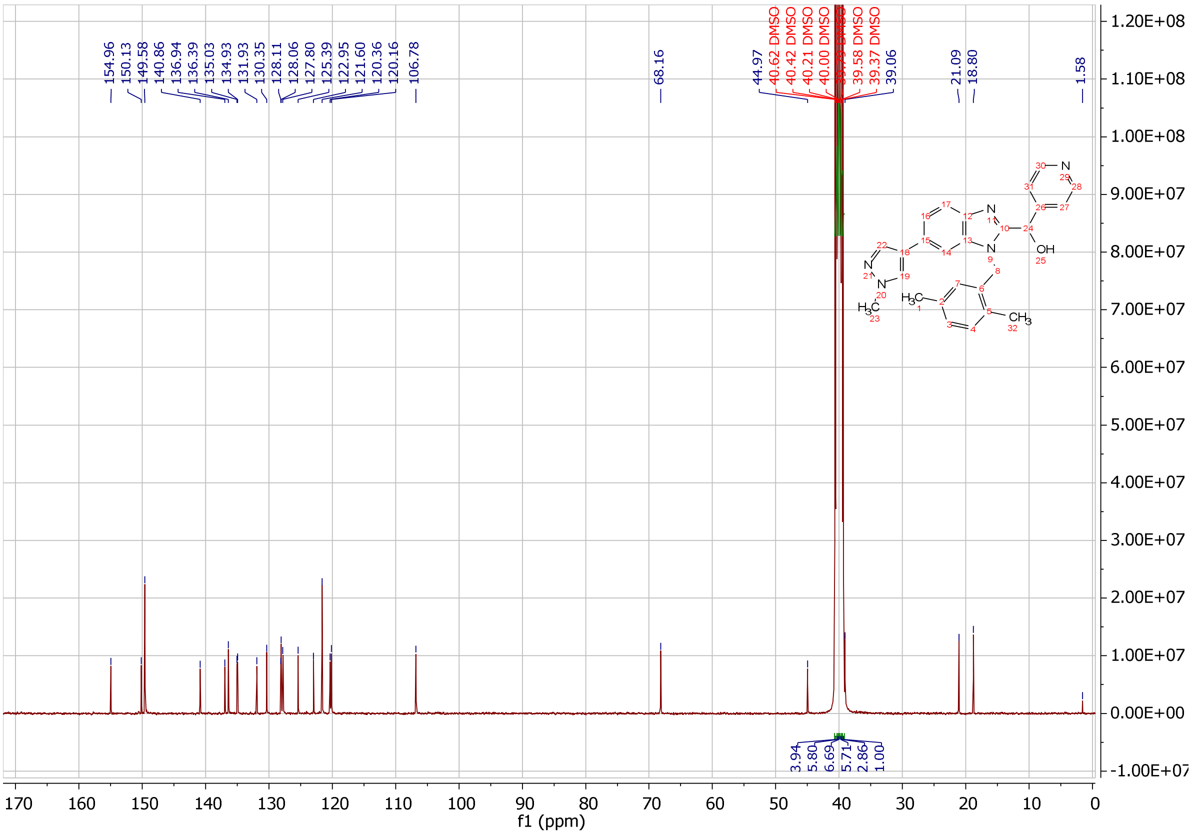

Supplementary Fig. 17: 13C NMR spectrum of UCB-9260 (101 MHz).

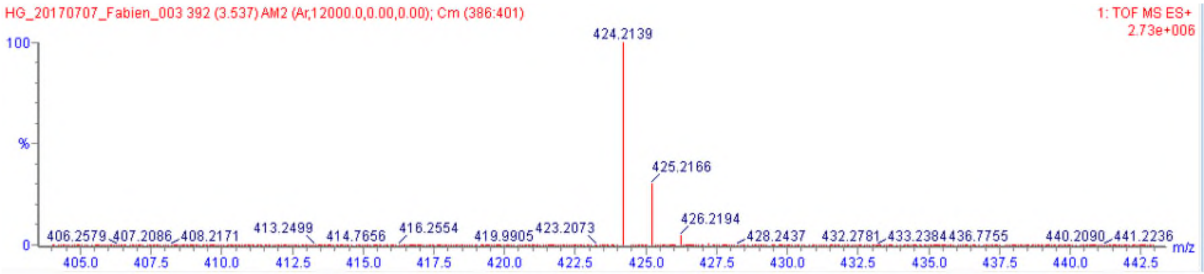

Supplementary Fig. 18: HRMS spectrum of UCB-9260.

Supplementary Table 1: Displacement of selected TNF residues involved in TNFR1 binding in the presence of compound

**Residue**	**Movement Å**
**H73**	**9.5**
**L75**	**6.3**
**T77**	**5.6**
**V91**	**8.3**
**I97**	**6.5**

An overlay of apo and UCB-**6876** bound human TNF (aligned through monomer C) was used to measure the displacement of monomer A. The degree of displacement (in Å) of selected residues on monomer A are shown (measurements taken on a-carbon atoms).

Supplementary Table 2: Theoretical and measured masses, MS monomer exchange

Species	H3 +6876	H2+M	H2+M+6876	H+2M	H+2M+6876	M +6876	M3
Molecular Weight (Da)	52577	51859	52125	51407	51673	51221	50995
Theoretical (m/z)	4780	4714	4746	4673	4698	4656	4632
Measured (m/z)	4780	4715	ND	4674	ND	4657	4633

Supplementary Table 3: Effect on the thermal stability of human TNF

**Sample**	**Tm (^{o} C)**<br>**(mean ± SD)**	**Tm difference**<br>**(hTNF + compound) - (hTNF+DMSO)**
hTNF	70.2 ± 0.4	No DMSO control
hTNF + DMSO (5%)	60.7 ± 0.1	-
hTNF + DMSO (5%) + UCB-**5307**	72.9 ± 0.6	12.2
hTNF + DMSO (5%) + UCB-**9260**	78.0 ± 0.7	17.3

Mean ± SD is taken from n=4 replicates within a single experiment. Thermal denaturation assay showing the increase in melting temperature (Tm) of human TNF (hTNF, generated at UCB) after the binding of UCB-**5307** and UCB-**9260**.

Supplementary Table 4: UCB-9260 in the mouse and human TNF neutrophilia models

UCB-9260 Dose mg/kg	Human TNF	Mouse TNF
	**Blood concentration 0.5h post dose total (free), nM**	**Blood concentration 0.5h post dose total (free), nM**
10	830 ± 184 (20 ± 4)	1,319 ± 88 (32 ± 2)
30	8,464 ± 2,454 (203 ± 59)	9,362 ± 947 (225 ± 23)
100	29,832 ± 3,891 (716 ± 93)	56,098 ± 24,416 (1,346 ± 586)
300	49,499 ± 4,608 (1,188 ± 111)	53,015 ± 5,015 (1,272 ± 120)

Mean of n=3 mice per group ± s.e.m. IC_{50} 202nM: human TNF and IC_{50} 95nM: mouse TNF in the HEK-293 NF-kB Reporter gene assays. Mouse free fraction (Fu) 2.4%. Source data are provided as a Source Data file.

Supplementary Table 5: UCB-9260 in the zymosan-induced neutrophilia model

UCB-9260 Dose mg/kg	Zymosan-induced neutrophil recruitment
	**Blood concentration 1h post dose (free), nM**
10	86 ± 15 (2 ± 0.4)
30	2,901 ± 272 (70 ± 7)
100	26,255 ± 4,103 (630 ± 98)
300	28,219 ± 5,059 (677 ± 121)

Mean of n=8 mice ± s.e.m. IC_{50} 120 nM. Mouse free fraction (Fu) 2.4%. Source data are provided as a Source Data file.

Supplementary Table 6: UCB-9260 in the CAIA model

UCB-9260 Dose mg/kg B.I.D.	Blood concentration total (free), nM		Cave post 1st dose total (free), nM
	**1h post dose**	**6h post dose**	
150	17,304 ± 4,151 (415 ± 100)	9,974 ± 4,203 (239 ± 101)	12,808 ± 3,070 (307 ± 74)

Mean of n = 9 mice ± s.e.m. Source data are provided as a Source Data file.

Supplementary Table 7: Crystallographic parameters for UCB-6876, -5307 and -9260

	**UCB-6876**	**UCB-5307**	**UCB-9260**
**PDB ID#**	**6OOY**	**6OOZ**	**6OP0**
**Data collection**			
Space group	$P2_12_12_1$ (19)	$P2_12_12_1$ (19)	$P2_12_12_1$ (19)
Cell dimensions			
a, *b*, *c* (Å)	54.53, 81.60, 92.26	53.55, 81.21, 93.40	54.17, 81.99, 94.17
α, β, γ (°)	90.0, 90.0, 90.0	90.0, 90.0, 90.0	90.0, 90.0, 90.0
Resolution (Å)	20.0-2.50 (2.57-2.50)	50.0-2.80 (2.87-2.80)	50.0-2.55 (2.62-2.55)
R_{sym} or R_{merge}	9.1 (49.0)	9.2 (52.7)	7.6 (52.2)
I / σI	13.40 (2.96)	15.0 (2.89)	18.54 (3.76)
Completeness (%)	99.3 (99.9)	99.4 (99.7)	99.4 (100.0)
Redundancy	3.6 (3.7)	4.3 (4.4)	6.3 (6.4)
**Refinement**			
Resolution (Å)	19.92-2.5 (2.59-2.50)	19.83-2.80 (2.87-2.80)	19.89-2.55 (2.62-2.55)
No. reflections	14,708 (1,052)	10,433 (728)	14,153 (914)
R_{work} / R_{free}	0.201 / 0.272 (0.244 / 0.299)	0.183 / 0.274 (0.252 / 0.408)	0.185 / 0.244 (0.287 / 0.474)
No. atoms			
Protein	3190	3162	3083
Ligand/ion	28	27	32
Water	83	44	67
B-factors			
Protein	24.52	39.11	35.95
Ligand/ion	7.97	29.12	26.01
Water	19.69	26.00	28.86
R.m.s. deviations			
Bond lengths (Å)	0.033	0.011	0.015
Bond angles (°)	1.76	1.54	1.43

*A single crystal was used for each structure discussed.
*Values in parentheses are for highest-resolution shell.
#PDB ID: 6OOY [https://www.rcsb.org/structure/6ooY]; 6OOZ [https://www.rcsb.org/structure/6ooY]; 6OP0 [https://www.rcsb.org/structure/6op0]
