# Supplementary material for: Small molecules that inhibit TNF signalling by stabilising an asymmetric form of the trimer
Source: Nat Commun. 2019 Dec 19;10:5795. doi: 10.1038/s41467-019-13616-1 (PMC6923382; doi:10.1038/s41467-019-13616-1)
Supplement: Supplementary file 3 — Description of Additional Supplementary Files [file 41467_2019_13616_MOESM3_ESM.docx]

**Description of Supplementary Files**

**File Name:** Supplementary Video 1

**Description:** *Crystal structure of how UCB-6876 binds to distorted homotrimer in the presence of MPD.* This movie was generated using Pymol. The crystal structures of TNF in the presence and absence of UCB-6876 were morphed. Particularly noticeable is the relative movement of the tyrosines in the centre. TNF is seen from above showing the symmetrical homotrimer before crystallisation with UCB-6876. Movement of the monomers can be seen from above and from the side. Space filling is then used to show the compound and MPD buried within the middle of the homotrimer.
