# Supplementary material for: Small molecules that inhibit TNF signalling by stabilising an asymmetric form of the trimer
Source: Nat Commun. 2019 Dec 19;10:5795. doi: 10.1038/s41467-019-13616-1 (PMC6923382; doi:10.1038/s41467-019-13616-1)
Supplement: Supplementary file 5 — Reporting Summary [file 41467_2019_13616_MOESM5_ESM.pdf]

nature research

Corresponding author(s): James O'Connell

Last updated by author(s): Aug 12, 2019

Reporting Summary

Nature Research wishes to improve the reproducibility of the work that we publish. This form provides structure for consistency and transparency in reporting. For further information on Nature Research policies, see Authors & Referees and the Editorial Policy Checklist.

Statistics

For all statistical analyses, confirm that the following items are present in the figure legend, table legend, main text, or Methods section.

n/a	Confirmed	
☐	☒	The exact sample size (*n*) for each experimental group/condition, given as a discrete number and unit of measurement
☐	☒	A statement on whether measurements were taken from distinct samples or whether the same sample was measured repeatedly
☐	☒	The statistical test(s) used AND whether they are one- or two-sided<br>*Only common tests should be described solely by name; describe more complex techniques in the Methods section.*
☒	☐	A description of all covariates tested
☒	☐	A description of any assumptions or corrections, such as tests of normality and adjustment for multiple comparisons
☐	☒	A full description of the statistical parameters including central tendency (e.g. means) or other basic estimates (e.g. regression coefficient) AND variation (e.g. standard deviation) or associated estimates of uncertainty (e.g. confidence intervals)
☒	☐	For null hypothesis testing, the test statistic (e.g. *F*, *t*, *r*) with confidence intervals, effect sizes, degrees of freedom and *P* value noted<br>*Give P values as exact values whenever suitable.*
☒	☐	For Bayesian analysis, information on the choice of priors and Markov chain Monte Carlo settings
☒	☐	For hierarchical and complex designs, identification of the appropriate level for tests and full reporting of outcomes
☒	☐	Estimates of effect sizes (e.g. Cohen's *d*, Pearson's *r*), indicating how they were calculated

Our web collection on statistics for biologists contains articles on many of the points above.

Software and code

Policy information about availability of computer code

Data collection

X-ray experiments experiments were performed on custom software for SSRL beamline 7-1 and ALS beamline 5.0.3

Data analysis

Additionally, Applied published crystallography tools:

Applied published crystallography tools:
Data reduction - XDS (Versions January 30, 2009, December 31, 2001, and July 4, 2012)
Data scaling - XSCALE (Versions January 30, 2009, December 31, 2001, and July 4, 2012)
Refinement - CCP4 program suite, Refmac5 (Version 5.5.0109 and 5.6.0117)
Validation - Molprobity
Validation - wwPDB validation service

Statistical tools
SAS 9.4, SAS Institute Inc., Cary, NC, USA

Molecular simulation software

FREAD ref.. 42	Open source code for structure
Maestro ref. 44	Proprietary Structure based design application from Schrodinger
AmberTools 1.4 ref. 45	Open source code for building and analysing molecular dynamics data
NAMD 2.7 ref. 47	Open source molecular dynamics application
ACEMD ref. 48	Proprietary molecular dynamics application from Acelera
Plumed 1.3 ref. 49	Open source molecular dynamics application
GROMACS ref. 52	Open source molecular dynamics application

Biacore software
Biacore T100 BIAevaluation software version 1.1

Biacore A100 BIAevaluation software version 1.1

Mass spectrometry software
Data was processed using ThermoFisher Protein Deconvolution 3.0 software

For manuscripts utilizing custom algorithms or software that are central to the research but not yet described in published literature, software must be made available to editors/reviewers. We strongly encourage code deposition in a community repository (e.g. GitHub). See the Nature Research guidelines for submitting code & software for further information.

Data

Policy information about availability of data

All manuscripts must include a data availability statement. This statement should provide the following information, where applicable:

- Accession codes, unique identifiers, or web links for publicly available datasets
- A list of figures that have associated raw data
- A description of any restrictions on data availability

The authors declare that [the/all other] data supporting the findings of this study are available within the paper [and its supplementary information files].

Field-specific reporting

Please select the one below that is the best fit for your research. If you are not sure, read the appropriate sections before making your selection.

☒ Life sciences ☐ Behavioural & social sciences ☐ Ecological, evolutionary & environmental sciences

For a reference copy of the document with all sections, see nature.com/documents/nr-reporting-summary-flat.pdf

Life sciences study design

All studies must disclose on these points even when the disclosure is negative.

Sample size	No sample size calculation was made in the current study. Sample sizes for animal studies based on standard protocols to enable statistic validity as shown in the text. Where n=1 data is used, data in the published experiment is supported by a number of experiments on compounds with the same mode of action not described in the text.
Data exclusions	No data excluded except in animal experiments where data only excluded as a consequence of technical issues e.g. blood contamination in lavage fluid
Replication	Where applied, all attempts at replication were successful and are included in the data analyses
Randomization	Cages of animals were randomized into groups at the start of each study.
Blinding	Investigators were not blinded to group allocation, but arthritis scores were independently checked

Reporting for specific materials, systems and methods

We require information from authors about some types of materials, experimental systems and methods used in many studies. Here, indicate whether each material, system or method listed is relevant to your study. If you are not sure if a list item applies to your research, read the appropriate section before selecting a response.

Materials & experimental systems

n/a	Involved in the study	
☐	☒	Antibodies
☐	☒	Eukaryotic cell lines
☒	☐	Palaeontology
☐	☒	Animals and other organisms
☒	☐	Human research participants
☒	☐	Clinical data

Methods

n/a	Involved in the study	
☒	☐	ChIP-seq
☒	☐	Flow cytometry
☒	☐	MRI-based neuroimaging

Antibodies

Antibodies used	List of antibodies used in publication.<br>Immunoprecipitation: anti-TNFR1 (polyclonal goat R&D AB-225-PB): Human TNF RI/TNFRSF1A Antibody<br>Control (polyclonal goat R&D AB-108-C): Normal Goat IgG Control<br>Western blots:<br>anti-RIP1 (BD-610459 1:250 dilution); detection with anti-mouse-HRP (Cell Signalling -7076 1:2000 dilution) : full name Mouse

	Anti-RIP Clone 38/RIP<br>anti-pNFkB (Cell Signalling -3033 1:1000 dilution); detection with anti-rabbit HRP (Cell Signalling-7074 1:2000 dilution) : full name Phospho-NF-κB p65 (Ser536) (93H1) Rabbit mAb<br>anti-GAPDH Cell Signalling- 5174 1:4000 dilution); detection with anti-rabbit HRP (Cell Signalling-7074 1:2000 dilution) : full name GAPDH (D16H11) XP® Rabbit mAb
Validation	All antibodies confirmed to be applicable for methods, as detailed in data sheets provided by commercial supplier

Eukaryotic cell lines

Policy information about cell lines

Cell line source(s)	HEK-BlueTM CD40L SEAP (secreted embryonic alkaline phosphatase) reporter cell line (InvivoGen, #hkb-CD40)<br>Jurkat (Clone 6E-1 , human acute T cell lymphoma suspension cell line – ATCC - TIB-152)<br>L929 cells (ECACC, catalogue #85011425)
Authentication	Authentication included in supplier data sheets
Mycoplasma contamination	All cell lines were negative for mycoplasma
Commonly misidentified lines<br>(See ICLAC register)	None used

Animals and other organisms

Policy information about studies involving animals; ARRIVE guidelines recommended for reporting animal research

Laboratory animals	Male Balb/c mice aged 6 -8 weeks were used in all in vivo studies
Wild animals	The study did not involve wild animals
Field-collected samples	The study did not involve samples collected from the field
Ethics oversight	All in vivo studies were reviewed by an internal Ethical Review Body (ERB) and conducted in accordance with the Animals (Scientific Procedures) Act 1986, EU Directive 2010/63/EU.

Note that full information on the approval of the study protocol must also be provided in the manuscript.
