# Supplementary material for: Small molecules that inhibit TNF signalling by stabilising an asymmetric form of the trimer
Source: Nat Commun. 2019 Dec 19;10:5795. doi: 10.1038/s41467-019-13616-1 (PMC6923382; doi:10.1038/s41467-019-13616-1)
Supplement: Supplementary file 2 — Peer Review File [file 41467_2019_13616_MOESM2_ESM.pdf]

Editorial Note: This manuscript has been previously reviewed at another journal that is not operating a transparent peer review scheme. The manuscript was considered suitable for publication without further review at Nature Communications.
